# Supplementary material for: Direct Consideration of Process History During Intensified Design of Experiments Planning Eases Interpretation of Mammalian Cell Culture Dynamics
Source: Bioengineering (Basel). 2025 Mar 19;12(3):319. doi: 10.3390/bioengineering12030319 (PMC11939677; doi:10.3390/bioengineering12030319)
Supplement: Supplementary file 1 [file bioengineering-12-00319-s001.zip › bioengineering-3409830-supplementary.pdf]

5. Supplementary Materials

5.1 Design Planning and Evaluation

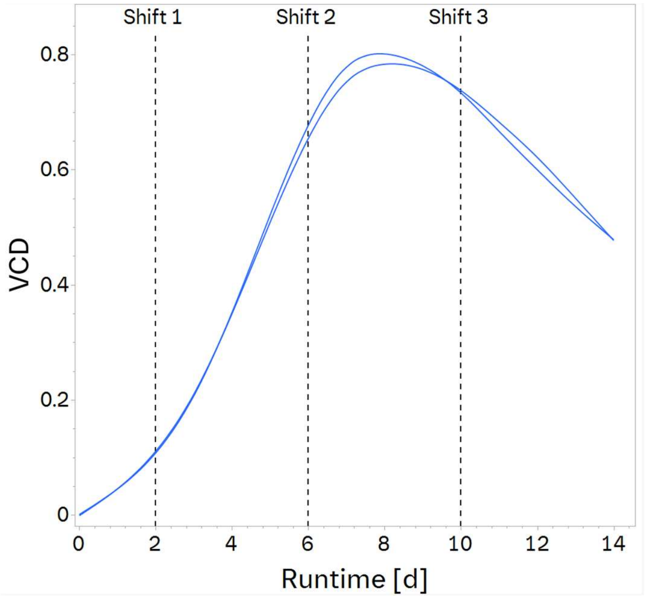

Figure S1. Pre-Cultivations executed to identify characteristic growth phases during the fed-batch cultivation. 2 cultivations were performed at standard operating conditions without intra-experimental factor shifts (T1 = T2 = T3 = 0= DO1 = DO2 = DO3 = 0). All other conditions during the cultivation were as described in the material and methods section. Smoothing has been applied for visualization.

 https://doi.org/10.3390/xxxxx

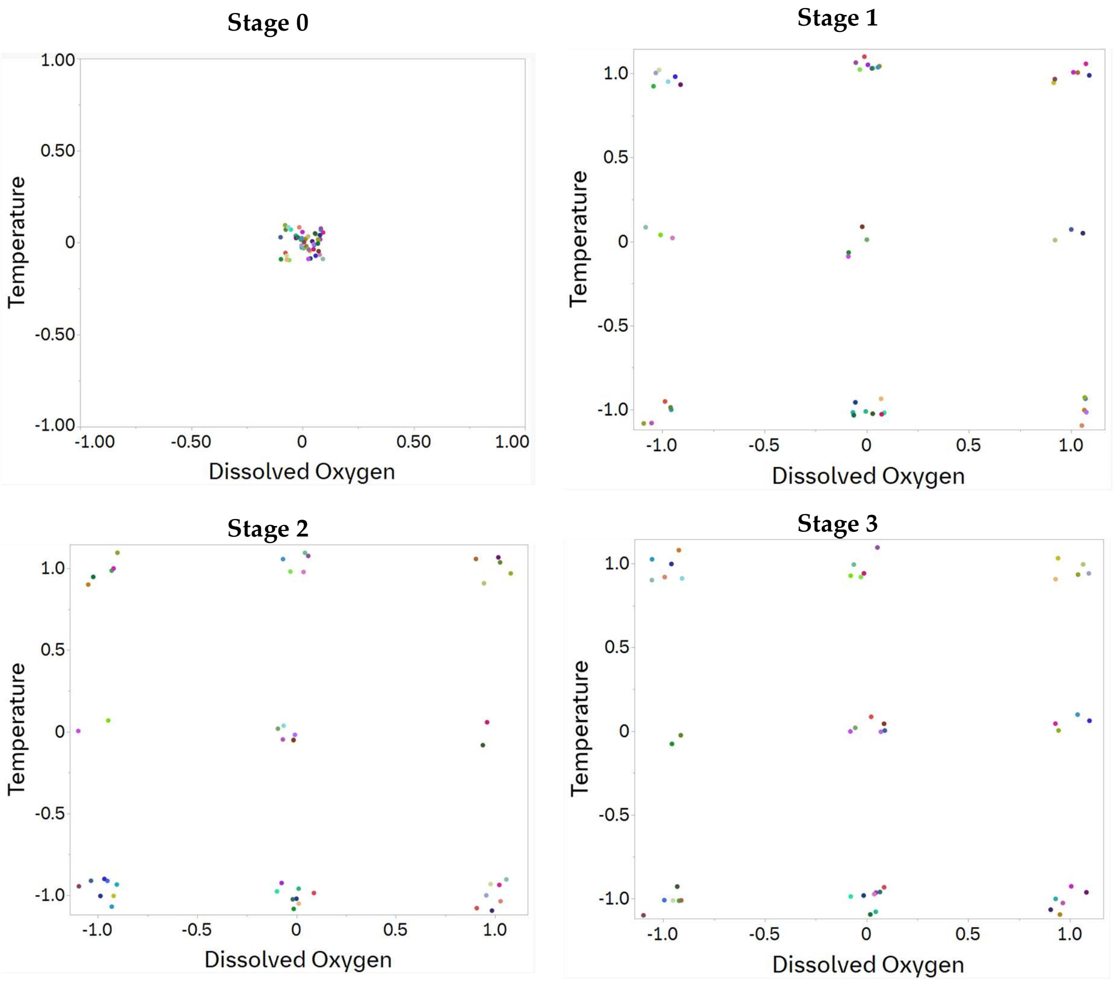

Figure S2. Visualization of the conducted iDoE for the investigation of the effect of the input factors temperature and dissolved oxygen over time during the mammalian cell culture process. Visualization of each stages factor settings for the bioreactors within the design space.

Table S1. Power at a signal to noise ratio of 2 and 3 (S/N=2, S/N =3). T1,2,3 = Temperature (day 2-6, 6-10, 10-14), DO1,2,3 = Dissolved oxygen (day 2-6, 6-10, 10-14), Init = Initial value of a response at the beginning of a stage.

Term	Standard Error	VIF	R_i^2	Power (SN=2)	Power (SN=3)
T1	0.1843	1.2874	0.2233	0.9993	1.0000
T2	0.1790	1.1750	0.1489	0.9996	1.0000
T3	0.1811	1.1697	0.1451	0.9995	1.0000
DO1	0.2094	1.2274	0.1853	0.9950	1.0000
DO2	0.2022	1.1852	0.1563	0.9969	1.0000
DO3	0.2060	1.2295	0.1866	0.9960	1.0000
T1:T2	0.2093	1.3976	0.2845	0.9950	1.0000
T1:T3	0.2029	1.2759	0.2162	0.9967	1.0000
T1:DO1	0.2507	1.3831	0.2770	0.9664	0.9999
T1:DO2	0.2555	1.4996	0.3332	0.9608	0.9998
T1:DO3	0.2248	1.2536	0.2023	0.9883	1.0000
T2:T3	0.2260	1.5158	0.3403	0.9876	1.0000
T2:DO1	0.2295	1.2599	0.2063	0.9854	1.0000

T2:DO2	0.2262	1.2784	0.2178	0.9874	1.0000
T2:DO3	0.2220	1.2233	0.1826	0.9898	1.0000
T3:DO1	0.2567	1.3826	0.2767	0.9593	0.9998
T3:DO2	0.2493	1.3674	0.2687	0.9680	0.9999
T3:DO3	0.2290	1.2050	0.1701	0.9857	1.0000
DO1:DO2	0.2362	1.3344	0.2506	0.9804	1.0000
DO1:DO3	0.2331	1.2482	0.1988	0.9829	1.0000
DO2:DO3	0.2331	1.2488	0.1992	0.9828	1.0000
T1^2	0.4217	1.4076	0.2896	0.9945	1.0000
T2^2	0.4242	1.4244	0.2980	0.9941	1.0000
T3^2	0.3854	1.3369	0.2520	0.9985	1.0000
DO1^2	0.4348	2.2052	0.5465	0.9920	1.0000
DO2^2	0.4322	2.1438	0.5335	0.9926	1.0000
DO3^2	0.3952	1.7930	0.4423	0.9978	1.0000

	Intercept	T1	T2	T3	DO1	DO2	DO3	T1:T2	T1:T3	T1:DO1	T1:DO2	T1:DO3	T2:T3	T2:DO1	T2:DO2	T2:DO3	T3:DO1	T3:DO2	T3:DO3	DO1:DO2	DO1:DO3	DO2:DO3	T1^2	T2^2	T3^2	DO1^2	DO2^2	DO3^2
Intercept	1.00	0.05	0.03	0.02	0.07	-0.01	-0.08	0.01	-0.09	0.09	0.13	-0.02	-0.09	0.03	-0.04	-0.04	0.06	0.04	0.09	-0.08	-0.10	0.04	-0.37	-0.35	-0.34	-0.15	-0.15	0.02
T1	0.05	1.00	-0.13	0.03	-0.01	0.05	-0.11	0.21	0.16	0.09	0.12	-0.05	0.13	0.01	-0.06	0.06	0.14	0.10	-0.08	0.16	-0.08	-0.07	0.08	-0.19	-0.16	-0.01	0.27	-0.09
T2	0.03	-0.13	1.00	-0.09	0.02	0.03	-0.08	-0.17	0.03	-0.04	-0.10	0.00	0.04	-0.04	0.02	-0.11	-0.21	-0.01	-0.09	-0.14	-0.05	0.01	-0.10	0.17	0.03	-0.07	-0.05	0.07
T3	0.02	0.03	-0.09	1.00	0.00	0.05	0.04	0.08	-0.19	0.00	0.12	-0.08	0.13	-0.17	0.07	-0.06	0.04	0.10	-0.01	-0.10	0.04	-0.07	0.10	-0.02	-0.02	-0.07	0.00	-0.01
DO1	0.07	-0.01	0.02	0.00	1.00	0.02	-0.01	-0.02	0.03	-0.10	0.13	-0.09	-0.18	0.08	-0.11	0.00	-0.04	-0.08	0.00	-0.03	0.05	-0.20	0.04	-0.08	0.05	0.09	-0.12	-0.07
DO2	-0.01	0.05	0.03	0.05	0.02	1.00	-0.02	-0.06	0.02	0.05	0.05	-0.09	-0.02	-0.17	0.17	-0.03	-0.11	0.05	-0.12	-0.04	-0.14	-0.01	0.01	0.02	0.06	-0.05	0.09	-0.12
DO3	-0.08	-0.11	-0.08	0.04	-0.01	-0.02	1.00	0.07	-0.10	-0.18	-0.11	-0.14	-0.05	-0.07	-0.01	0.15	-0.11	-0.15	0.06	-0.23	0.03	-0.04	-0.01	-0.01	0.11	0.02	0.06	-0.09
T1:T2	0.01	0.21	-0.17	0.08	-0.02	-0.06	0.07	1.00	-0.11	0.06	0.00	-0.07	0.22	0.00	-0.12	-0.08	0.15	-0.09	0.13	-0.14	0.00	0.03	0.02	-0.17	-0.19	0.11	0.19	0.04
T1:T3	-0.09	0.16	0.03	-0.19	0.03	0.02	-0.10	-0.11	1.00	0.02	-0.03	0.01	-0.10	0.10	-0.18	0.19	0.02	-0.07	-0.01	0.23	-0.08	0.00	0.01	0.05	-0.03	0.02	0.09	0.05
T1:DO1	0.09	0.09	-0.04	0.00	-0.10	0.05	-0.18	0.06	0.02	1.00	0.16	0.16	0.17	0.17	-0.08	-0.03	0.32	0.28	0.05	0.12	-0.11	0.16	0.00	-0.17	-0.04	0.10	-0.04	0.06
T1:DO2	0.13	0.12	-0.10	0.12	0.13	0.05	-0.11	0.00	-0.03	0.16	1.00	-0.02	-0.07	-0.13	0.14	0.07	0.25	0.29	-0.01	0.05	0.21	-0.22	0.19	-0.22	-0.16	0.09	0.03	-0.12
T1:DO3	-0.02	-0.05	0.00	-0.08	-0.09	-0.09	-0.14	-0.07	0.01	0.16	-0.02	1.00	0.12	0.08	0.04	0.04	0.08	0.09	0.18	0.20	0.00	0.02	-0.09	-0.06	0.18	0.15	-0.18	0.01
T2:T3	-0.09	0.13	0.04	0.13	-0.18	-0.02	-0.05	0.22	-0.10	0.17	-0.07	0.12	1.00	0.00	-0.03	-0.11	0.17	0.14	-0.07	-0.03	-0.04	-0.04	-0.07	-0.16	-0.07	0.17	0.13	0.17
T2:DO1	0.03	0.01	-0.04	-0.17	0.08	-0.17	-0.07	0.00	0.10	0.17	-0.13	0.08	0.00	1.00	-0.01	-0.06	0.19	-0.02	0.08	0.05	-0.14	0.14	-0.12	0.00	0.07	0.09	-0.08	0.03
T2:DO2	-0.04	-0.06	0.02	0.07	-0.11	0.17	-0.01	-0.12	-0.18	-0.08	0.14	0.04	-0.03	-0.01	1.00	-0.10	-0.04	0.18	-0.11	0.00	0.13	-0.10	0.01	0.01	0.16	0.03	-0.02	-0.14
T2:DO3	-0.04	0.06	-0.11	-0.06	0.00	-0.03	0.15	-0.08	0.19	-0.03	0.07	0.04	-0.11	-0.06	-0.10	1.00	0.04	-0.09	0.12	0.15	0.07	-0.07	0.17	-0.06	-0.05	-0.03	0.07	-0.11
T3:DO1	0.06	0.14	-0.21	0.04	-0.04	-0.11	-0.11	0.15	0.02	0.32	0.25	0.08	0.17	0.19	-0.04	0.04	1.00	0.22	0.09	0.15	0.03	-0.04	0.00	-0.21	-0.04	0.10	0.09	0.02
T3:DO2	0.04	0.10	-0.01	0.10	-0.08	0.05	-0.15	-0.09	-0.07	0.28	0.29	0.09	0.14	-0.02	0.18	-0.09	0.22	1.00	-0.06	0.14	-0.02	-0.10	0.04	-0.16	-0.01	0.18	-0.07	0.00
T3:DO3	0.09	-0.08	-0.09	-0.01	0.00	-0.12	0.06	0.13	-0.01	0.05	-0.01	0.18	-0.07	0.08	-0.11	0.12	0.09	-0.06	1.00	-0.04	0.06	0.07	-0.04	-0.14	0.06	0.10	-0.16	0.06
DO1:DO2	-0.08	0.16	-0.14	-0.10	-0.03	-0.04	-0.23	-0.14	0.23	0.12	0.05	0.20	-0.03	0.05	0.00	0.15	0.15	0.14	-0.04	1.00	-0.11	-0.06	0.09	0.03	0.00	0.08	0.02	-0.08
DO1:DO3	-0.10	-0.08	-0.05	0.04	0.05	-0.14	0.03	0.00	-0.08	-0.11	0.21	0.00	-0.04	-0.14	0.13	0.07	0.03	-0.02	0.06	-0.11	1.00	-0.03	0.23	-0.11	0.05	0.02	-0.01	-0.08
DO2:DO3	0.04	-0.07	0.01	-0.07	-0.20	-0.01	-0.04	0.03	0.00	0.16	-0.22	0.02	-0.04	0.14	-0.10	-0.07	-0.04	-0.10	0.07	-0.06	-0.03	1.00	0.00	-0.02	-0.01	-0.18	0.05	0.09
T1^2	-0.37	0.08	-0.10	0.10	0.04	0.01	-0.01	0.02	0.01	0.00	0.19	-0.09	-0.07	-0.12	0.01	0.17	0.00	0.04	-0.04	0.09	0.23	0.00	1.00	-0.22	-0.26	0.07	0.10	-0.26
T2^2	-0.35	-0.19	0.17	-0.02	-0.08	0.02	-0.01	-0.17	0.05	-0.17	-0.22	-0.06	-0.16	0.00	0.01	-0.06	-0.21	-0.16	-0.14	0.03	-0.11	-0.02	-0.22	1.00	-0.04	-0.15	-0.13	-0.03
T3^2	-0.34	-0.16	0.03	-0.02	0.05	0.06	0.11	-0.19	-0.03	-0.04	-0.16	0.18	-0.07	0.07	0.16	-0.05	-0.04	-0.01	0.06	0.00	0.05	-0.01	-0.26	-0.04	1.00	0.05	-0.10	-0.08
DO1^2	-0.15	-0.01	-0.07	-0.07	0.09	-0.05	0.02	0.11	0.02	0.10	0.09	0.15	0.17	0.09	0.03	-0.03	0.10	0.18	0.10	0.08	0.02	-0.18	0.07	-0.15	0.05	1.00	-0.42	-0.29
DO2^2	-0.15	0.27	-0.05	0.00	-0.12	0.09	0.06	0.19	0.09	-0.04	0.03	-0.18	0.13	-0.08	-0.02	0.07	0.09	-0.07	-0.16	0.02	-0.01	0.05	0.10	-0.13	-0.10	-0.42	1.00	-0.21
DO3^2	0.02	-0.09	0.07	-0.01	-0.07	-0.12	-0.09	0.04	0.05	0.06	-0.12	0.01	0.17	0.03	-0.14	-0.11	0.02	0.00	0.06	-0.08	-0.08	0.09	-0.26	-0.03	-0.08	-0.29	-0.21	1.00

Figure S3. Correlation matrix

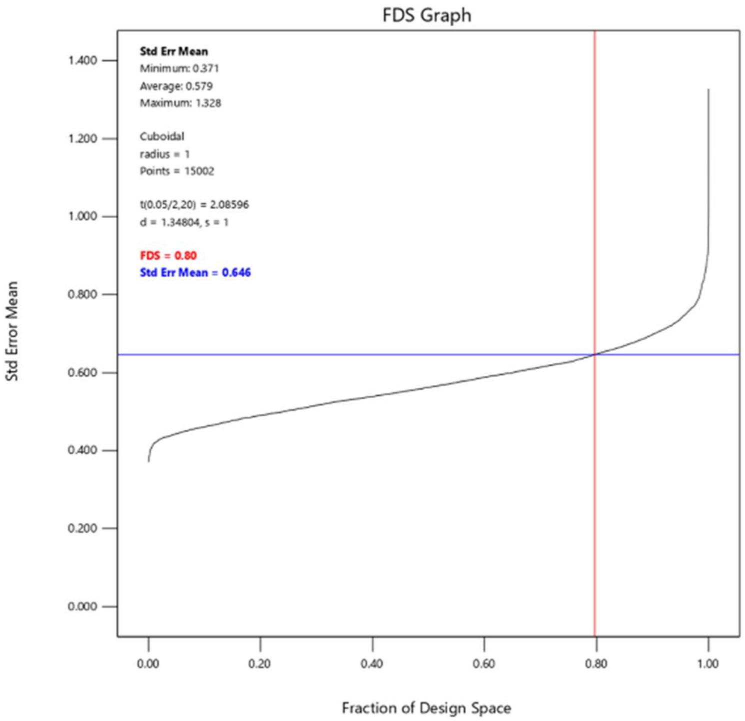

Figure S4. Fraction design space plot highlighting the prediction variance over 80% of the designspace.

5.2. One-Factor-One-Column: Stage-Wise Ordinary Least Squares Modeling

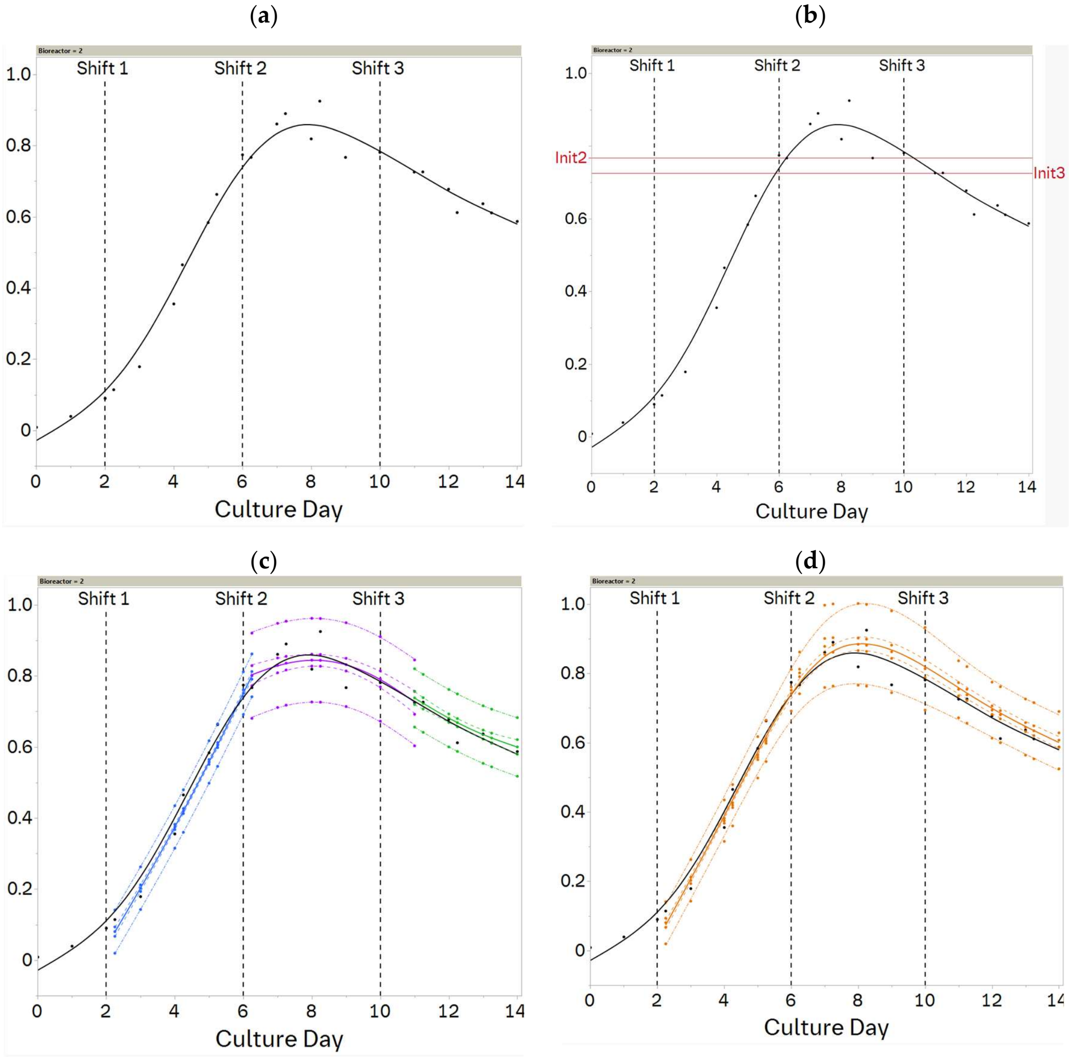

Figure S5. Stage-wise modeling approach for iDoE data. (a) Model complexity considered in the modeling of each respective stage, including the model used to predict the stage 3 Init for titer (b) Measured viable cell density during the fed-batch process for one bioreactor. (c) Determination of the Init values for each bioreactor as the initial response value at the beginning of stage 2 (Init2) and stage 3 (Init3) (d) Separate stage-wise regression models are fit (CI/PI = dashed lines). In stage 1 culture duration is considered as additional factor, in stage 2 and stage 3 Init is additionally considered (e) Concatenated regression model of stages 1, 2 and 3 (CI/PI = dashed lines), Shift times of factor settings at day 2, 6 and 10 are indicated by dashed lines.

Table S2. Summary statistics for stage-wise one-factor-one-column models for investigated responses.

Response	Stage	Number of Parameters	R^2	R^2_{adj}	R^2_{pred}	RASE	RMSE	RMSE (PRESS)	Prob > F
VCD	1	14	0.980	0.979	0.978	0.030	0.030	0.031	<.0001
VCD	2	17	0.992	0.992	0.992	0.058	0.059	0.061	<.0001
VCD	3	17	0.995	0.995	0.994	0.040	0.041	0.042	<.0001
Viability	1	10	0.711	0.704	0.695	0.008	0.008	0.008	<.0001
Viability	2	16	0.997	0.997	0.997	0.045	0.046	0.048	<.0001
Viability	3	16	0.992	0.992	0.991	0.053	0.054	0.056	<.0001

IVCD	1	10	0.987	0.987	0.986	0.007	0.007	0.007	<.0001
IVCD	2	17	0.994	0.994	0.993	0.030	0.030	0.031	<.0001
IVCD	3	20	1.000	1.000	1.000	0.013	0.014	0.014	<.0001
Titer	1+2	6	0.943	0.938	0.914	0.047	0.049	0.057	<.0001
Titer	3	13	0.997	0.997	0.997	0.032	0.033	0.034	<.0001

Table S3. Final one-factor-one-column stage-wise models. Polynomials are centered. Runtime1, Runtime2, Runtime3 indicate stage-wise rebased runtime with the first measurement of a respective stage being rebased to 0.

Stage	Response	Term	Estimate	Std. Error	t Ratio	Prob>|t|	Lower 95%	Upper 95%
1	VCD	Intercept	0.032	0.008	3.908	0.000	0.016	0.048
1	VCD	T1	0.122	0.002	68.460	0.000	0.119	0.126
1	VCD	DO1	-0.010	0.002	-4.698	0.000	-0.014	-0.006
1	VCD	Runtime1	0.145	0.003	48.285	0.000	0.139	0.151
1	VCD	(T1-0.05291):(T1-0.05291)	-0.015	0.004	-3.982	0.000	-0.023	-0.008
1	VCD	(T1-0.05291):(DO1+0.00265)	-0.006	0.002	-2.632	0.009	-0.011	-0.002
1	VCD	(DO1+0.00265):(DO1+0.00265)	-0.006	0.003	-1.923	0.055	-0.012	0.000
1	VCD	(T1-0.05291):(Runtime1-2.28571)	0.059	0.001	42.353	0.000	0.056	0.062
1	VCD	(DO1+0.00265):(Runtime1-2.28571)	-0.007	0.002	-4.501	0.000	-0.010	-0.004
1	VCD	(Runtime1-2.28571):(Runtime1-2.28571)	0.005	0.001	5.303	0.000	0.003	0.007
1	VCD	(T1-0.05291):(T1-0.05291):(Runtime1-2.28571)	-0.019	0.003	-6.556	0.000	-0.025	-0.013
1	VCD	(T1-0.05291):(DO1+0.00265):(Runtime1-2.28571)	-0.003	0.002	-1.854	0.065	-0.007	0.000
1	VCD	(DO1+0.00265):(DO1+0.00265):(Runtime1-2.28571)	-0.004	0.002	-1.762	0.079	-0.009	0.001
1	Viability	Intercept	1.008	0.002	512.249	0.000	1.004	1.012
1	Viability	T1	-0.004	0.000	-8.778	0.000	-0.005	-0.003
1	Viability	DO1	-0.003	0.001	-6.035	0.000	-0.004	-0.002
1	Viability	Runtime1	-0.006	0.001	-8.544	0.000	-0.007	-0.005
1	Viability	(T1-0.05291):(T1-0.05291)	-0.006	0.001	-5.935	0.000	-0.008	-0.004
1	Viability	(T1-0.05291):(Runtime1-2.28571)	-0.005	0.000	-12.446	0.000	-0.005	-0.004
1	Viability	(DO1+0.00265):(Runtime1-2.28571)	-0.002	0.000	-5.482	0.000	-0.003	-0.001
1	Viability	(Runtime1-2.28571):(Runtime1-2.28571)	-0.003	0.000	-12.342	0.000	-0.004	-0.003
1	Viability	(T1-0.05291):(T1-0.05291):(Runtime1-2.28571)	-0.003	0.001	-3.514	0.000	-0.004	-0.001
1	IVCD	Intercept	-0.019	0.001	-20.154	0.000	-0.021	-0.017
1	IVCD	T1	0.026	0.000	62.579	0.000	0.025	0.027
1	IVCD	DO1	0.000	0.000	-0.358	0.721	-0.001	0.001
1	IVCD	Runtime1	0.045	0.000	149.920	0.000	0.044	0.045
1	IVCD	(T1-0.04167):DO1	-0.002	0.001	-4.191	0.000	-0.003	-0.001
1	IVCD	(T1-0.04167):(Runtime1-2.25)	0.014	0.000	43.892	0.000	0.013	0.015
1	IVCD	DO1:(Runtime1-2.25)	-0.001	0.000	-1.432	0.153	-0.001	0.000
1	IVCD	(Runtime1-2.25):(Runtime1-2.25)	0.008	0.000	31.862	0.000	0.007	0.008
1	IVCD	(T1-0.04167):DO1:(Runtime1-2.25)	-0.001	0.000	-2.616	0.009	-0.002	0.000

2	VCD	T2	0.021	0.004	5.222	0.000	0.013	0.029
2	VCD	DO2	-0.006	0.004	-1.600	0.110	-0.014	0.001
2	VCD	Init2_VCD	0.961	0.013	72.190	0.000	0.935	0.987
2	VCD	Runtime2	0.052	0.005	10.644	0.000	0.042	0.062
2	VCD	(T2+0.16316):(T2+0.16316)	-0.002	0.008	-0.284	0.777	-0.017	0.013
2	VCD	(DO2+0.01842):(DO2+0.01842)	0.017	0.007	2.546	0.011	0.004	0.030
2	VCD	(T2+0.16316):(Init2_VCD-0.56829)	-0.116	0.018	-6.456	0.000	-0.152	-0.081
2	VCD	(Init2_VCD-0.56829):(Init2_VCD-0.56829)	-0.014	0.109	-0.128	0.898	-0.229	0.201
2	VCD	(T2+0.16316):(Runtime2-2.10526)	0.019	0.003	6.914	0.000	0.013	0.024
2	VCD	(DO2+0.01842):(Runtime2-2.10526)	-0.001	0.003	-0.207	0.836	-0.006	0.005
2	VCD	(Init2_VCD-0.56829)*(Runtime2-2.10526)	-0.138	0.011	-12.868	0.000	-0.160	-0.117
2	VCD	(Runtime2-2.10526)*(Runtime2-2.10526)	-0.014	0.001	-9.596	0.000	-0.017	-0.011
2	VCD	(T2+0.16316):(T2+0.16316):(Runtime2-2.10526)	-0.019	0.005	-3.918	0.000	-0.029	-0.010
2	VCD	(DO2+0.01842):(DO2+0.01842):(Runtime2-2.10526)	-0.011	0.004	-2.372	0.018	-0.019	-0.002
2	VCD	(T2+0.16316):(Init2_VCD-0.56829):(Runtime2-2.10526)	-0.072	0.012	-5.949	0.000	-0.095	-0.048
2	VCD	(Init2_VCD-0.56829):(Init2_VCD-0.56829):(Runtime2-2.10526)	-0.410	0.074	-5.510	0.000	-0.556	-0.263
2	Viability	T2	-0.018	0.003	-5.617	0.000	-0.025	-0.012
2	Viability	DO2	-0.005	0.003	-1.615	0.107	-0.011	0.001
2	Viability	Init2_Viability	1.064	0.009	122.142	0.000	1.046	1.081
2	Viability	Runtime2	-0.056	0.003	-18.551	0.000	-0.062	-0.050
2	Viability	(T2+0.16667):(T2+0.16667)	-0.014	0.006	-2.275	0.023	-0.026	-0.002
2	Viability	(DO2+0.02083):(DO2+0.02083)	0.014	0.005	2.856	0.005	0.004	0.024
2	Viability	(T2+0.16667):(Init2_Viability-0.9661)	0.487	0.138	3.528	0.000	0.216	0.758
2	Viability	(Init2_Viability-0.9661):(Init2_Viability-0.9661)	2.165	4.628	0.468	0.640	-6.935	11.265
2	Viability	(T2+0.16667):(Runtime2-2.09375)	-0.010	0.002	-5.203	0.000	-0.014	-0.006
2	Viability	(DO2+0.02083):(Runtime2-2.09375)	-0.001	0.002	-0.564	0.573	-0.005	0.003
2	Viability	(Init2_Viability-0.9661):(Runtime2-2.09375)	0.648	0.090	7.164	0.000	0.470	0.826
2	Viability	(Runtime2-2.09375):(Runtime2-2.09375)	-0.010	0.001	-9.088	0.000	-0.012	-0.008
2	Viability	(DO2+0.02083):(DO2+0.02083):(Runtime2-2.09375)	0.009	0.003	2.799	0.005	0.003	0.015
2	Viability	(T2+0.16667):(Init2_Viability-0.9661):(Runtime2-2.09375)	0.166	0.093	1.787	0.075	-0.017	0.348
2	Viability	(Init2_Viability-0.9661):(Init2_Viability-0.9661):(Runtime2-2.09375)	13.943	3.392	4.111	0.000	7.273	20.612
2	IVCD	T2	0.020	0.002	9.345	0.000	0.016	0.024
2	IVCD	DO2	-0.002	0.002	-1.103	0.271	-0.006	0.002
2	IVCD	Init2_IVCD	1.591	0.025	63.513	0.000	1.542	1.640
2	IVCD	Runtime2	0.053	0.002	22.511	0.000	0.048	0.057
2	IVCD	(T2+0.16667):(T2+0.16667)	-0.020	0.004	-4.796	0.000	-0.028	-0.012

2	IVCD	(DO2+0.02083):(DO2+0.02083)	-0.016	0.003	-4.771	0.000	-0.022	-0.009
2	IVCD	(T2+0.16667):(Init2_IVCD-0.18471)	-0.201	0.036	-5.624	0.000	-0.271	-0.131
2	IVCD	(DO2+0.02083):(Init2_IVCD-0.18471)	-0.074	0.040	-1.826	0.069	-0.153	0.006
2	IVCD	(Init2_IVCD-0.18471):(Init2_IVCD-0.18471)	-9.154	0.902	-10.146	0.000	-10.928	-7.380
2	IVCD	(T2+0.16667):(Runtime2-2.09375)	0.001	0.001	0.469	0.639	-0.002	0.003
2	IVCD	(DO2+0.02083):(Runtime2-2.09375)	-0.001	0.001	-0.719	0.473	-0.004	0.002
2	IVCD	(Init2_IVCD-0.18471):(Runtime2-2.09375)	0.428	0.021	19.961	0.000	0.386	0.470
2	IVCD	(T2+0.16667):(T2+0.16667):(Runtime2-2.09375)	0.010	0.003	3.978	0.000	0.005	0.015
2	IVCD	(DO2+0.02083):(DO2+0.02083)*(Runtime2-2.09375)	0.008	0.002	3.745	0.000	0.004	0.013
2	IVCD	(T2+0.16667)*(Init2_IVCD-0.18471)*(Runtime2-2.09375)	-0.057	0.024	-2.404	0.017	-0.104	-0.010
2	IVCD	(Init2_IVCD-0.18471):(Init2_IVCD-0.18471):(Runtime2-2.09375)	3.292	0.607	5.424	0.000	2.099	4.486
3	VCD	T3	-0.018	0.003	-6.669	0.000	-0.024	-0.013
3	VCD	DO3	0.007	0.003	2.554	0.011	0.002	0.013
3	VCD	Init3_VCD	0.916	0.010	90.327	0.000	0.896	0.936
3	VCD	Runtime3	-0.039	0.004	-11.015	0.000	-0.046	-0.032
3	VCD	(T3+0.08333):(T3+0.08333)	0.023	0.005	4.583	0.000	0.013	0.033
3	VCD	(T3+0.08333):(DO3-0.02083)	0.010	0.003	3.090	0.002	0.004	0.016
3	VCD	(DO3-0.02083):(DO3-0.02083)	0.014	0.005	3.094	0.002	0.005	0.023
3	VCD	(T3+0.08333):(Init3_VCD-0.59428)	-0.050	0.020	-2.544	0.011	-0.089	-0.011
3	VCD	(Init3_VCD-0.59428):(Init3_VCD-0.59428)	0.365	0.110	3.334	0.001	0.150	0.581
3	VCD	(T3+0.08333):(Runtime3-1.39286)	-0.013	0.003	-5.041	0.000	-0.019	-0.008
3	VCD	(DO3-0.02083):(Runtime3-1.39286)	0.004	0.003	1.398	0.163	-0.002	0.010
3	VCD	(Init3_VCD-0.59428):(Runtime3-1.39286)	-0.188	0.022	-8.380	0.000	-0.232	-0.144
3	VCD	(Runtime3-1.39286):(Runtime3-1.39286)	0.006	0.002	2.305	0.022	0.001	0.011
3	VCD	(T3+0.08333):(DO3-0.02083):(Runtime3-1.39286)	0.008	0.003	2.327	0.021	0.001	0.014
3	VCD	(T3+0.08333):(Init3_VCD-0.59428):(Runtime3-1.39286)	-0.089	0.021	-4.228	0.000	-0.131	-0.048
3	VCD	(Init3_VCD-0.59428):(Init3_VCD-0.59428):(Runtime3-1.39286)	-0.446	0.150	-2.975	0.003	-0.741	-0.151
3	Viability	T3	-0.026	0.004	-6.965	0.000	-0.033	-0.018
3	Viability	DO3	0.005	0.004	1.252	0.212	-0.003	0.012
3	Viability	Init3_Viability	1.048	0.013	81.050	0.000	1.022	1.073
3	Viability	Runtime3	-0.133	0.006	-21.814	0.000	-0.145	-0.121
3	Viability	(T3+0.08333):(T3+0.08333)	0.007	0.007	0.990	0.323	-0.007	0.020
3	Viability	(T3+0.08333):(DO3-0.02083)	0.007	0.004	1.572	0.117	-0.002	0.015
3	Viability	(T3+0.08333):(Init3_Viability-0.71362)	0.166	0.034	4.864	0.000	0.099	0.233
3	Viability	(Init3_Viability-0.71362):(Init3_Viability-0.71362)	0.561	0.185	3.026	0.003	0.196	0.926
3	Viability	(T3+0.08333):(Runtime3-1.39286)	-0.013	0.004	-3.534	0.000	-0.020	-0.006

3	Viability	(DO3-0.02083):(Runtime3-1.39286)	0.006	0.004	1.597	0.111	-0.001	0.014
3	Viability	(Init3_Viability-0.71362):(Runtime3-1.39286)	0.145	0.027	5.324	0.000	0.092	0.199
3	Viability	(T3+0.08333):(T3+0.08333):(Runtime3-1.39286)	0.019	0.007	2.838	0.005	0.006	0.032
3	Viability	(T3+0.08333):(DO3-0.02083):(Runtime3-1.39286)	0.008	0.004	1.953	0.052	0.000	0.017
3	Viability	(T3+0.08333):(Init3_Viability-0.71362):(Runtime3-1.39286)	0.128	0.034	3.770	0.000	0.061	0.194
3	Viability	(Init3_Viability-0.71362):(Init3_Viability-0.71362):(Runtime3-1.39286)	1.015	0.200	5.084	0.000	0.622	1.407
3	IVCD	T3	-0.005	0.001	-5.700	0.000	-0.007	-0.003
3	IVCD	DO3	0.003	0.001	2.817	0.005	0.001	0.005
3	IVCD	Init3_IVCD	1.057	0.004	270.234	0.000	1.049	1.065
3	IVCD	Runtime3	0.060	0.002	36.368	0.000	0.057	0.064
3	IVCD	(T3+0.08333):(T3+0.08333)	0.001	0.002	0.307	0.759	-0.003	0.004
3	IVCD	(T3+0.08333):(DO3-0.02083)	0.004	0.001	3.438	0.001	0.002	0.006
3	IVCD	(DO3-0.02083):(DO3-0.02083)	-0.001	0.002	-0.912	0.362	-0.004	0.002
3	IVCD	(T3+0.08333):(Init3_IVCD-0.5544)	-0.036	0.006	-6.396	0.000	-0.047	-0.025
3	IVCD	(DO3-0.02083):(Init3_IVCD-0.5544)	0.018	0.006	2.846	0.005	0.006	0.030
3	IVCD	(Init3_IVCD-0.5544):(Init3_IVCD-0.5544)	-0.659	0.051	-12.991	0.000	-0.759	-0.559
3	IVCD	(T3+0.08333):(Runtime3-1.39286)	-0.004	0.001	-4.001	0.000	-0.005	-0.002
3	IVCD	(DO3-0.02083):(Runtime3-1.39286)	0.002	0.001	1.854	0.065	0.000	0.004
3	IVCD	(Init3_IVCD-0.5544):(Runtime3-1.39286)	0.066	0.005	12.119	0.000	0.055	0.076
3	IVCD	(Runtime3-1.39286):(Runtime3-1.39286)	-0.004	0.001	-4.736	0.000	-0.006	-0.002
3	IVCD	(T3+0.08333):(T3+0.08333):(Runtime3-1.39286)	0.005	0.002	3.148	0.002	0.002	0.009
3	IVCD	(T3+0.08333):(DO3-0.02083):(Runtime3-1.39286)	0.002	0.001	2.122	0.035	0.000	0.004
3	IVCD	(DO3-0.02083):(DO3-0.02083):(Runtime3-1.39286)	0.003	0.002	2.137	0.033	0.000	0.006
3	IVCD	(T3+0.08333):(Init3_IVCD-0.5544):(Runtime3-1.39286)	-0.017	0.006	-3.010	0.003	-0.028	-0.006
3	IVCD	(Init3_IVCD-0.5544):(Init3_IVCD-0.5544):(Runtime3-1.39286)	-0.084	0.055	-1.516	0.131	-0.192	0.025
1+2	Init3_Titer	Intercept	0.386	0.007	54.916	0.000	0.372	0.399
1+2	Init3_Titer	T1	0.204	0.003	71.646	0.000	0.199	0.210
1+2	Init3_Titer	T2	0.012	0.003	3.562	0.000	0.005	0.018
1+2	Init3_Titer	DO1	-0.057	0.003	-17.398	0.000	-0.063	-0.051
1+2	Init3_Titer	DO2	-0.011	0.003	-3.442	0.001	-0.017	-0.005
1+2	Init3_Titer	(T1-0.04167):(T1-0.04167)	-0.047	0.006	-7.711	0.000	-0.059	-0.035
1+2	Init3_Titer	(T2+0.16667):(T2+0.16667)	-0.006	0.006	-0.981	0.328	-0.019	0.006
1+2	Init3_Titer	(T1-0.04167):DO1	0.022	0.004	6.197	0.000	0.015	0.030
1+2	Init3_Titer	DO1:DO1	-0.027	0.006	-4.301	0.000	-0.040	-0.015
1+2	Init3_Titer	(T2+0.16667):(DO2+0.02083)	-0.007	0.003	-2.034	0.043	-0.014	0.000
1+2	Init3_Titer	(DO2+0.02083):(DO2+0.02083)	0.022	0.006	3.417	0.001	0.009	0.035

3	Titer	T3	0.005	0.003	1.690	0.093	-0.001	0.011
3	Titer	DO3	-0.003	0.003	-0.887	0.376	-0.009	0.003
3	Titer	Init3_Titer	1.151	0.012	99.020	0.000	1.128	1.174
3	Titer	Runtime3	0.128	0.003	39.343	0.000	0.122	0.134
3	Titer	(T3+0.08333):(T3+0.08333)	-0.022	0.006	-3.931	0.000	-0.033	-0.011
3	Titer	(T3+0.08333):(DO3-0.02083)	0.012	0.004	3.223	0.002	0.004	0.019
3	Titer	(T3+0.08333):(Init3_Titer-0.34744)	-0.027	0.014	-1.933	0.055	-0.055	0.001
3	Titer	(DO3-0.02083):(Init3_Titer-0.34744)	0.029	0.017	1.704	0.090	-0.005	0.063
3	Titer	(Init3_Titer-0.34744):(Init3_Titer-0.34744)	-1.275	0.094	-13.584	0.000	-1.460	-1.090
3	Titer	(Init3_Titer-0.34744):(Runtime3-1.5)	0.126	0.012	10.676	0.000	0.103	0.149
3	Titer	(Runtime3-1.5):(Runtime3-1.5)	-0.006	0.002	-2.714	0.007	-0.011	-0.002
3	Titer	(Init3_Titer-0.34744):(Init3_Titer-0.34744):(Runtime3-1.5)	-0.527	0.076	-6.918	0.000	-0.677	-0.377

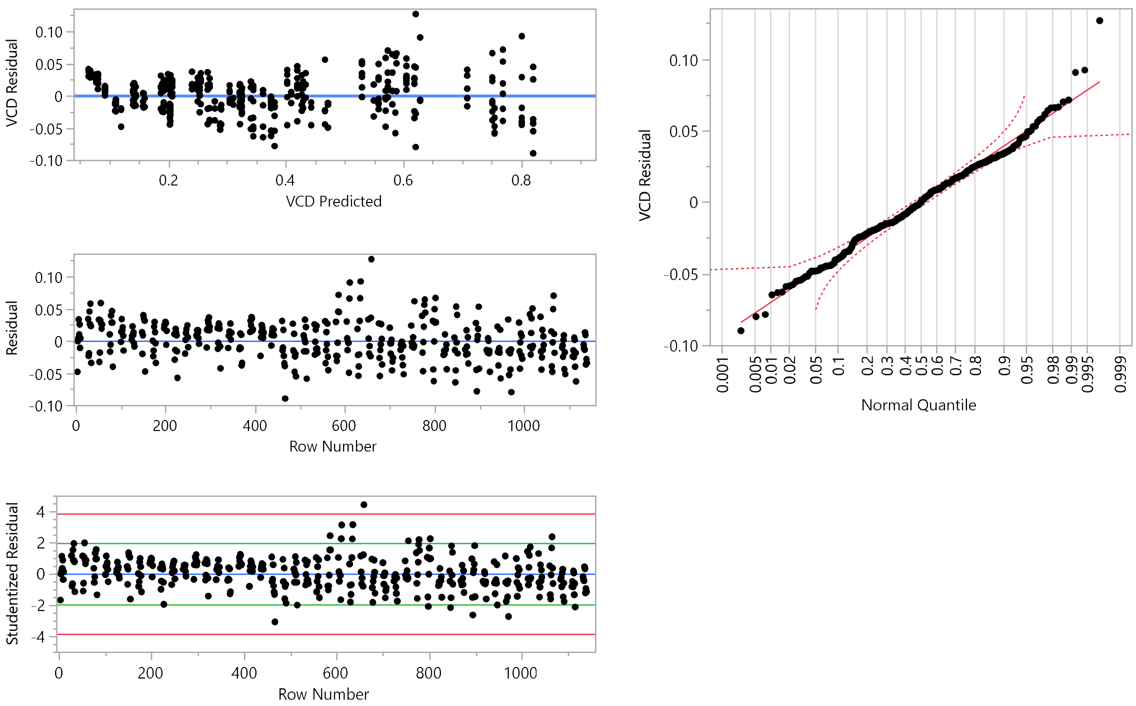

Figure S6. Residual plots for the Stage 1 model of viable cell density (VCD) using the one-factor-one-column stage-wise (OFOC-SW) modeling approach. Externally studentized residuals with 95% simultaneous limits (Bonferroni) in red, individual limits in green. Lilliefors confidence bounds in the normal quantile plot as dashed lines.

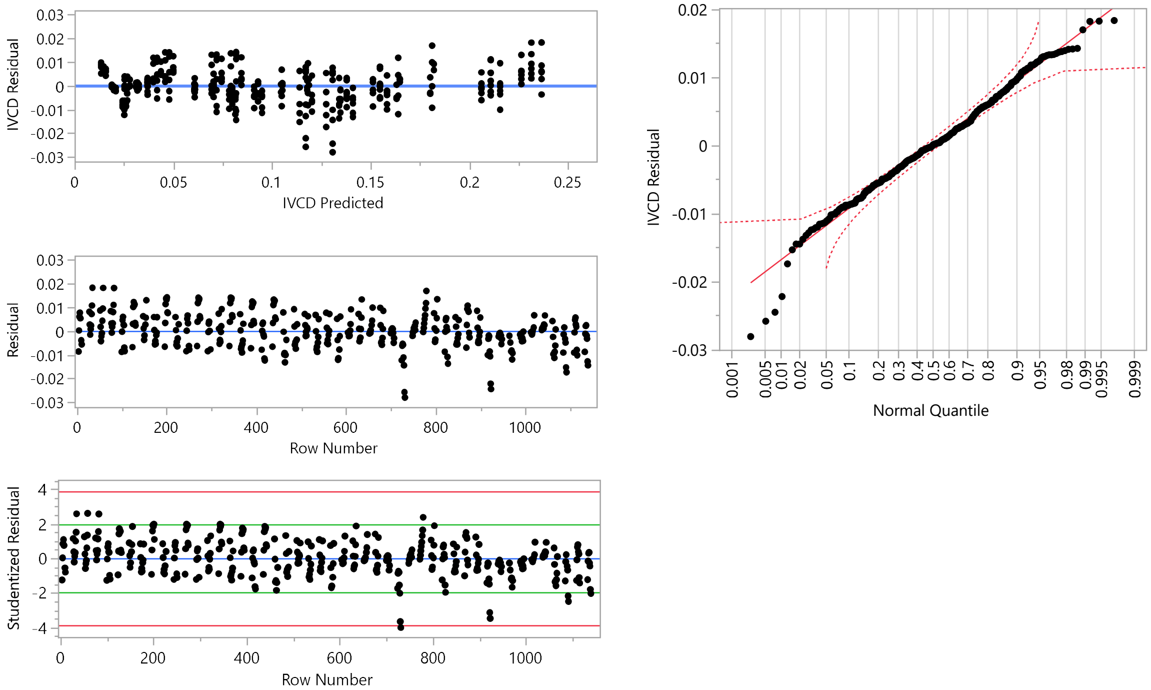

Figure S7. Residual plots for the Stage 1 model of integral viable cell density (IVCD) using the one-factor-one-column stage-wise (OFOC-SW) modeling approach. Externally studentized residuals with 95% simultaneous limits (Bonferroni) in red, individual limits in green. Lilliefors confidence bounds in the normal quantile plot as dashed lines.

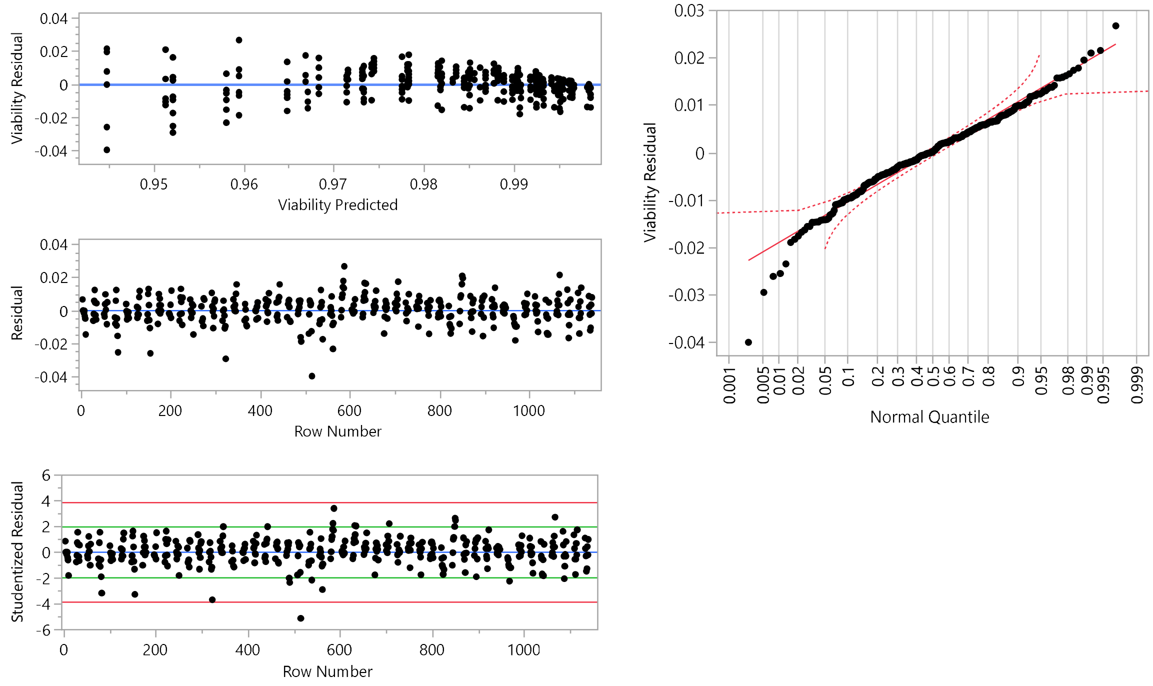

Figure S8. Residual plots for the Stage 1 model of viability using the one-factor-one-column stage-wise (OFOC-SW) modeling approach. Externally studentized residuals with 95% simultaneous limits (Bonferroni) in red, individual limits in green. Lilliefors confidence bounds in the normal quantile plot as dashed lines.

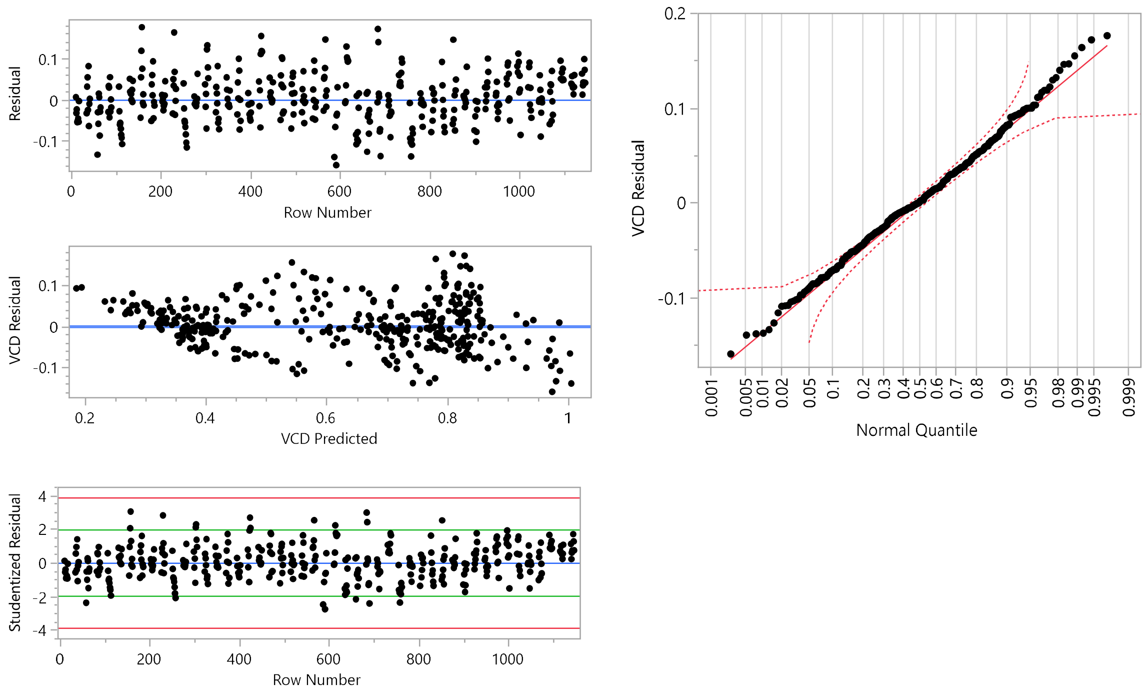

Figure S9. Residual plots for the Stage 2 model of viable cell density (VCD) using the one-factor-one-column stage-wise (OFOC-SW) modeling approach. Externally studentized residuals with 95% simultaneous limits (Bonferroni) in red, individual limits in green. Lilliefors confidence bounds in the normal quantile plot as dashed lines.

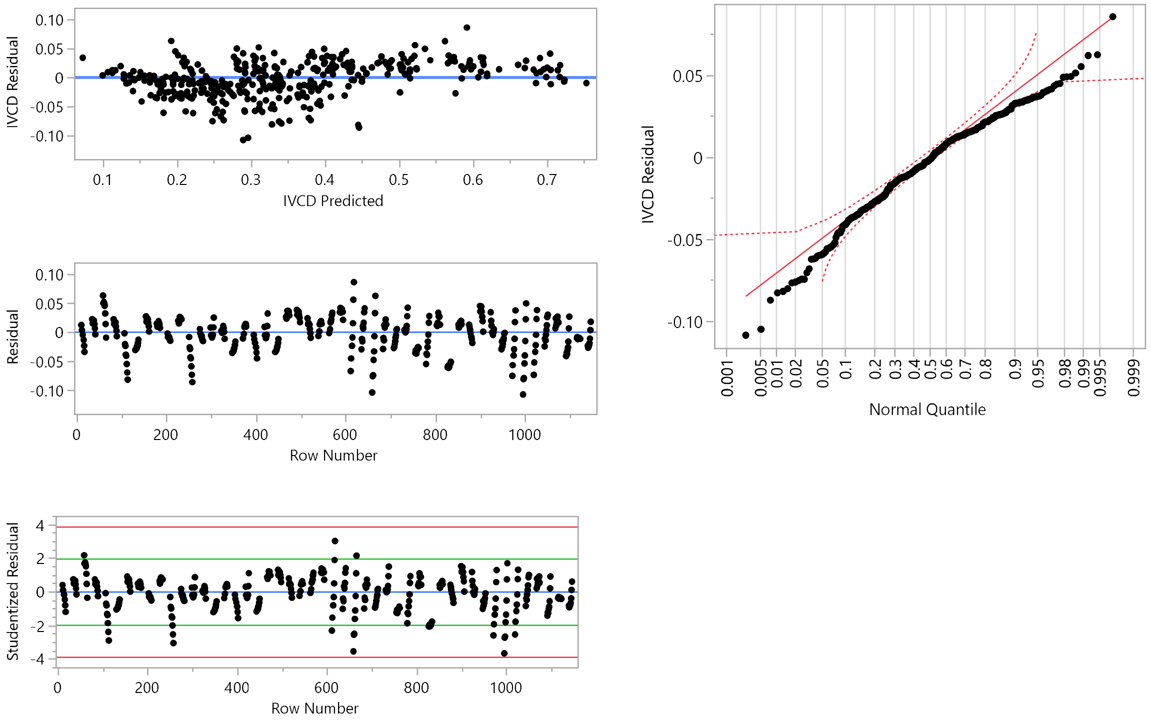

Figure S10. Residual plots for the Stage 2 model of integral viable cell density (IVCD) using the one-factor-one-column stage-wise (OFOC-SW) modeling approach. Externally studentized residuals

with 95% simultaneous limits (Bonferroni) in red, individual limits in green. Lilliefors confidence bounds in the normal quantile plot as dashed lines.

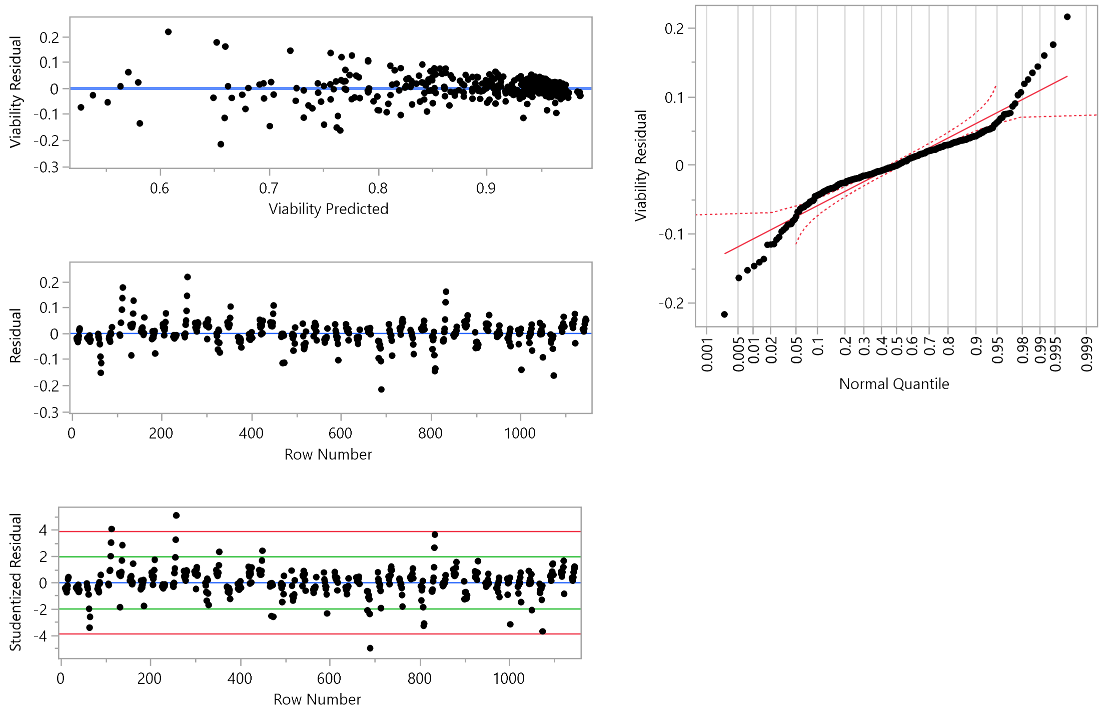

Figure S11. Residual plots for the Stage 2 model of viability using the one-factor-one-column stage-wise (OFOC-SW) modeling approach. Externally studentized residuals with 95% simultaneous limits (Bonferroni) in red, individual limits in green. Lilliefors confidence bounds in the normal quantile plot as dashed lines.

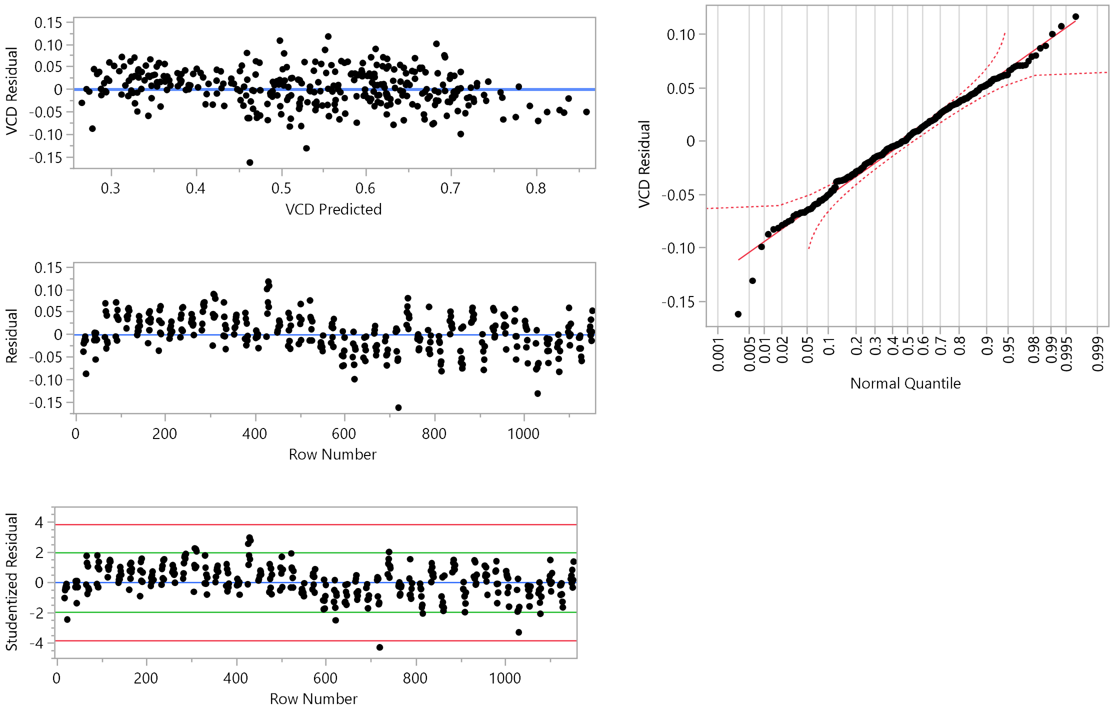

Figure S12. Residual plots for the Stage 3 model of viable cell density (VCD) using the one-factor-one-column stage-wise (OFOC-SW) modeling approach. Externally studentized residuals with 95% simultaneous limits (Bonferroni) in red, individual limits in green. Lilliefors confidence bounds in the normal quantile plot as dashed lines.

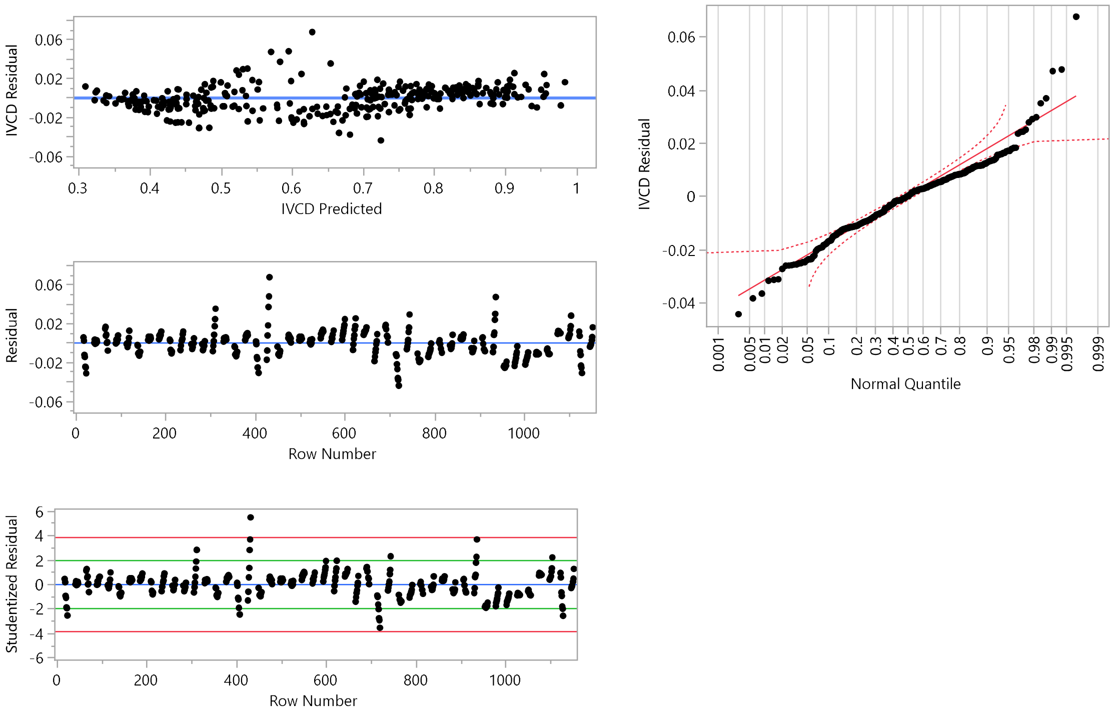

Figure S13. Residual plots for the Stage 3 model of integral viable cell density (IVCD) using the one-factor-one-column stage-wise (OFOC-SW) modeling approach. Externally studentized residuals with 95% simultaneous limits (Bonferroni) in red, individual limits in green. Lilliefors confidence bounds in the normal quantile plot as dashed lines.

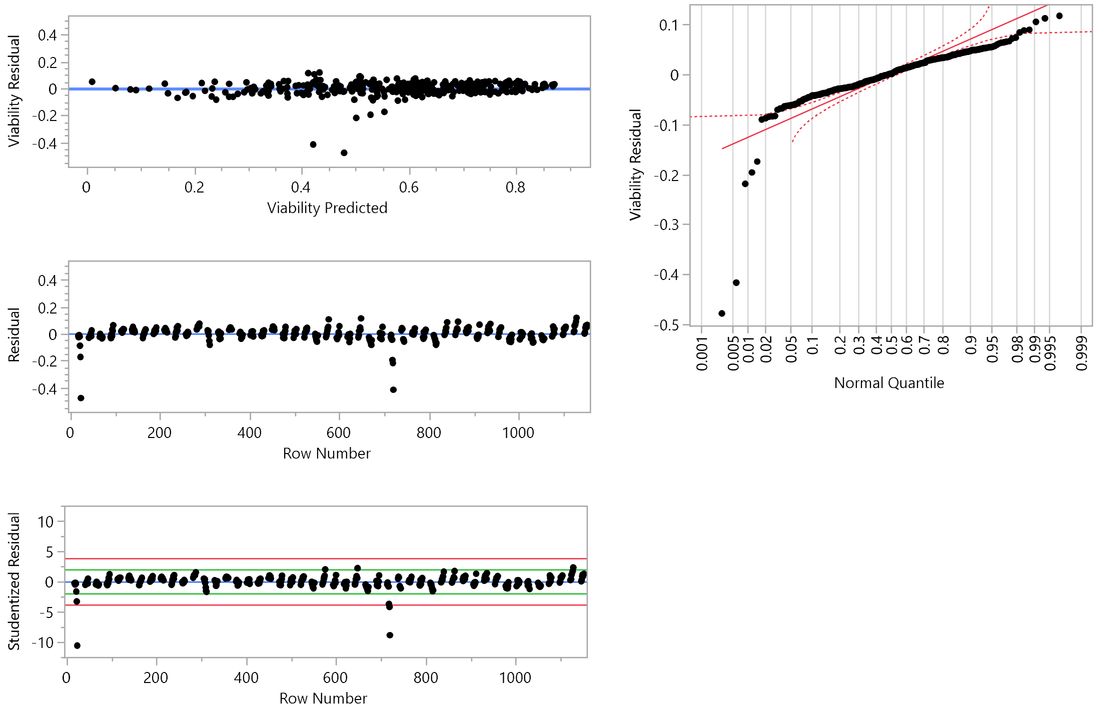

Figure S14. Residual plots for the Stage 3 model of viability using the one-factor-one-column stage-wise (OFOC-SW) modeling approach. Externally studentized residuals with 95% simultaneous limits (Bonferroni) in red, individual limits in green. Lilliefors confidence bounds in the normal quantile plot as dashed lines.

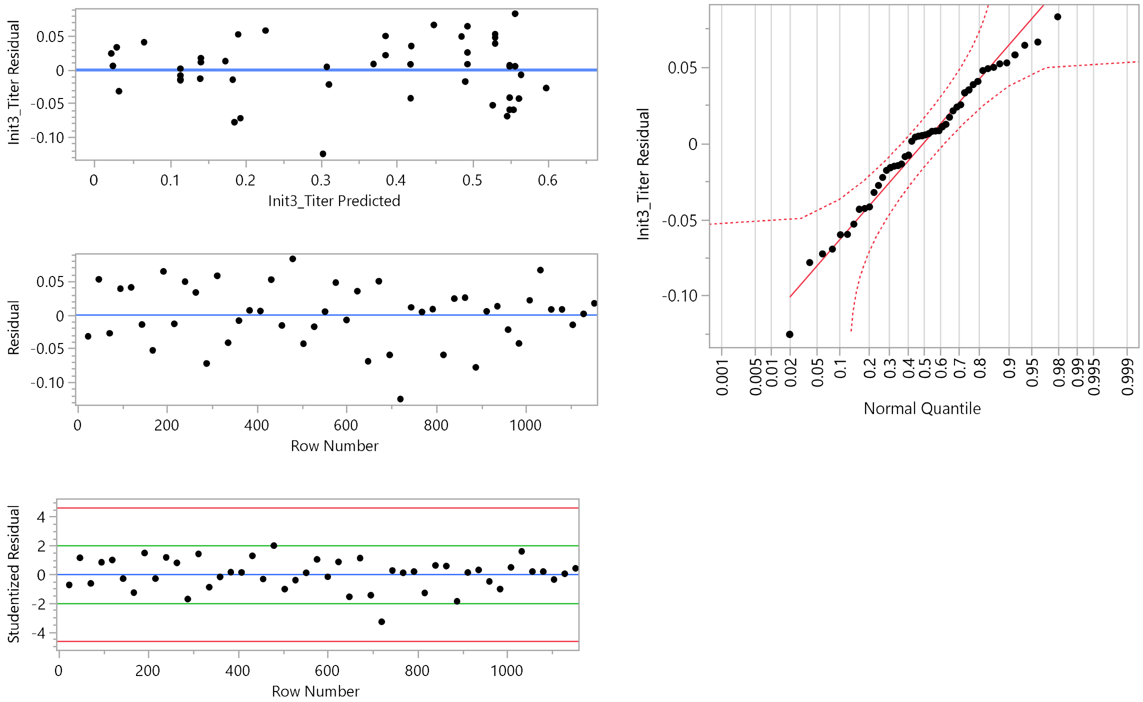

Figure S15. Residual plots for the Init3_Titer model of viability using the one-factor-one-column stage-wise (OFOC-SW) modeling approach. Externally studentized residuals with 95%

simultaneous limits (Bonferroni) in red, individual limits in green. Lilliefors confidence bounds in the normal quantile plot as dashed lines.

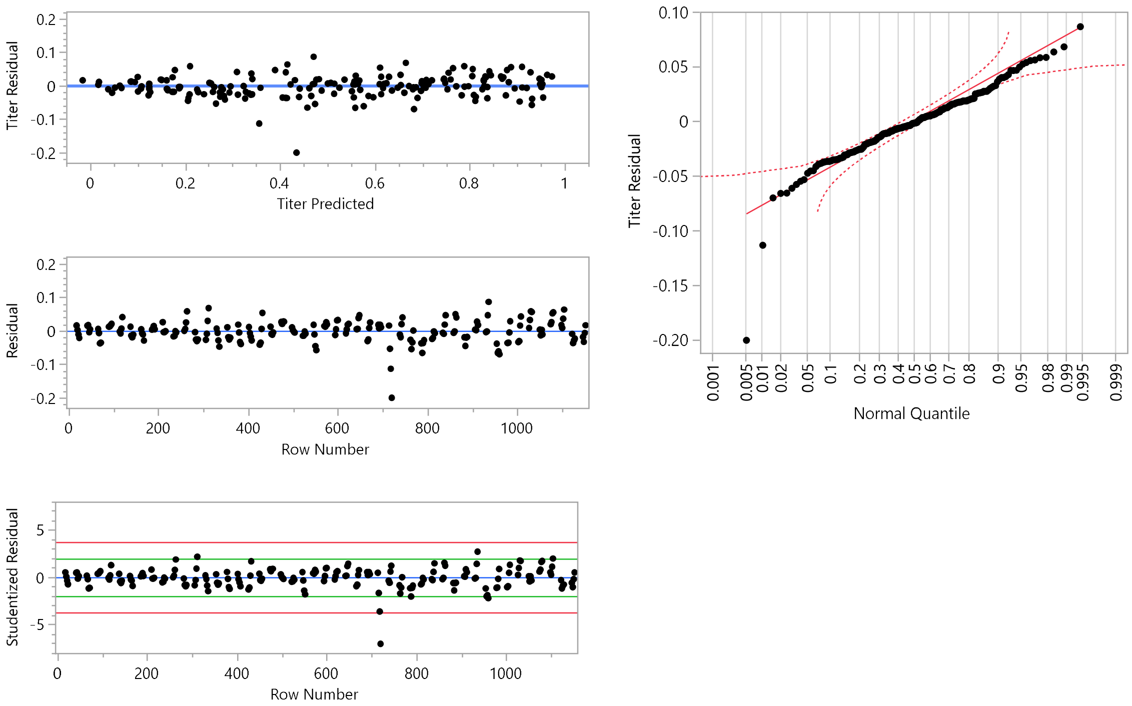

Figure S16. Residual plots for the Stage 3 model of titer using the one-factor-one-column stage-wise (OFOC-SW) modeling approach. Externally studentized residuals with 95% simultaneous limits (Bonferroni) in red, individual limits in green. Lilliefors confidence bounds in the normal quantile plot as dashed lines.

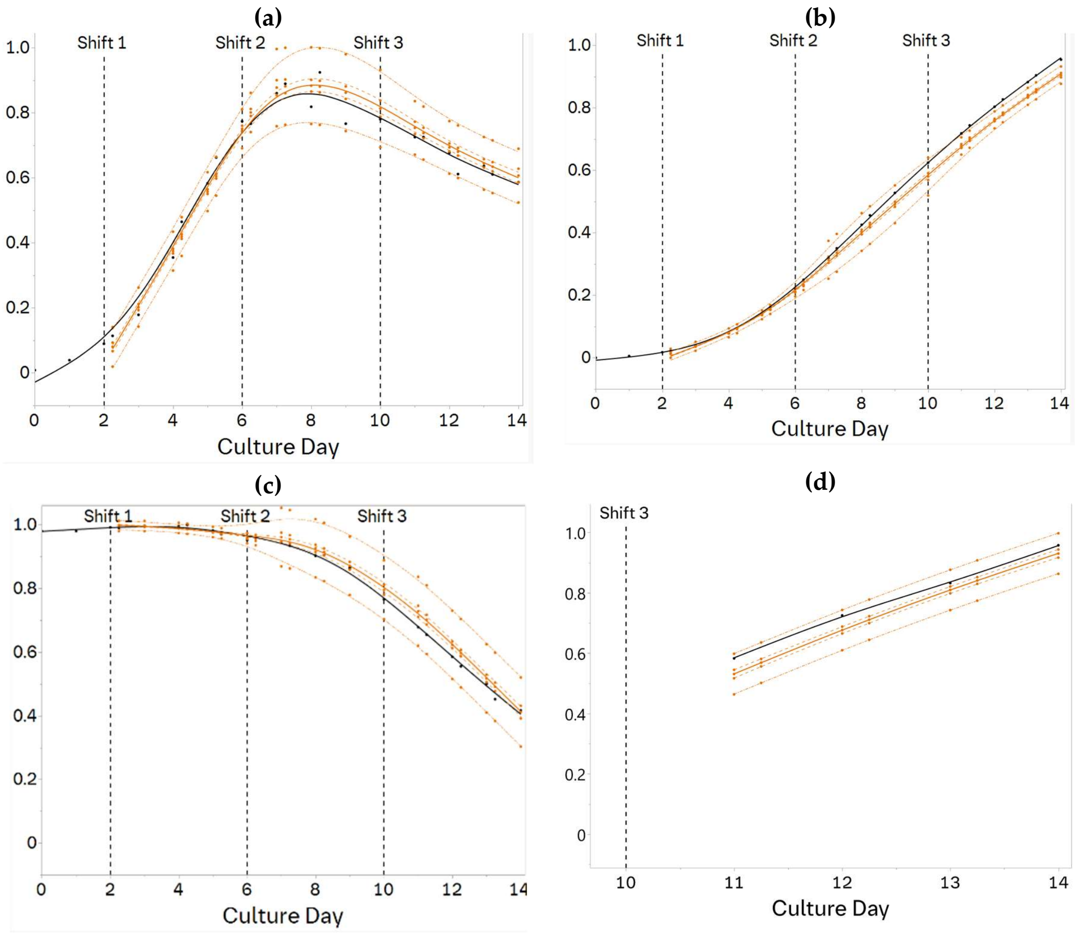

Figure S17. Predictions of the stage-wise analysis for the functional responses viable cell density (**a**), 84
integral viable cell density (**b**), viability (**c**) and titer (**d**) (CI/PI = dashed lines). Shift times of factor 85
settings at day 2, 6 and 10 are indicated by dashed lines. 86

87

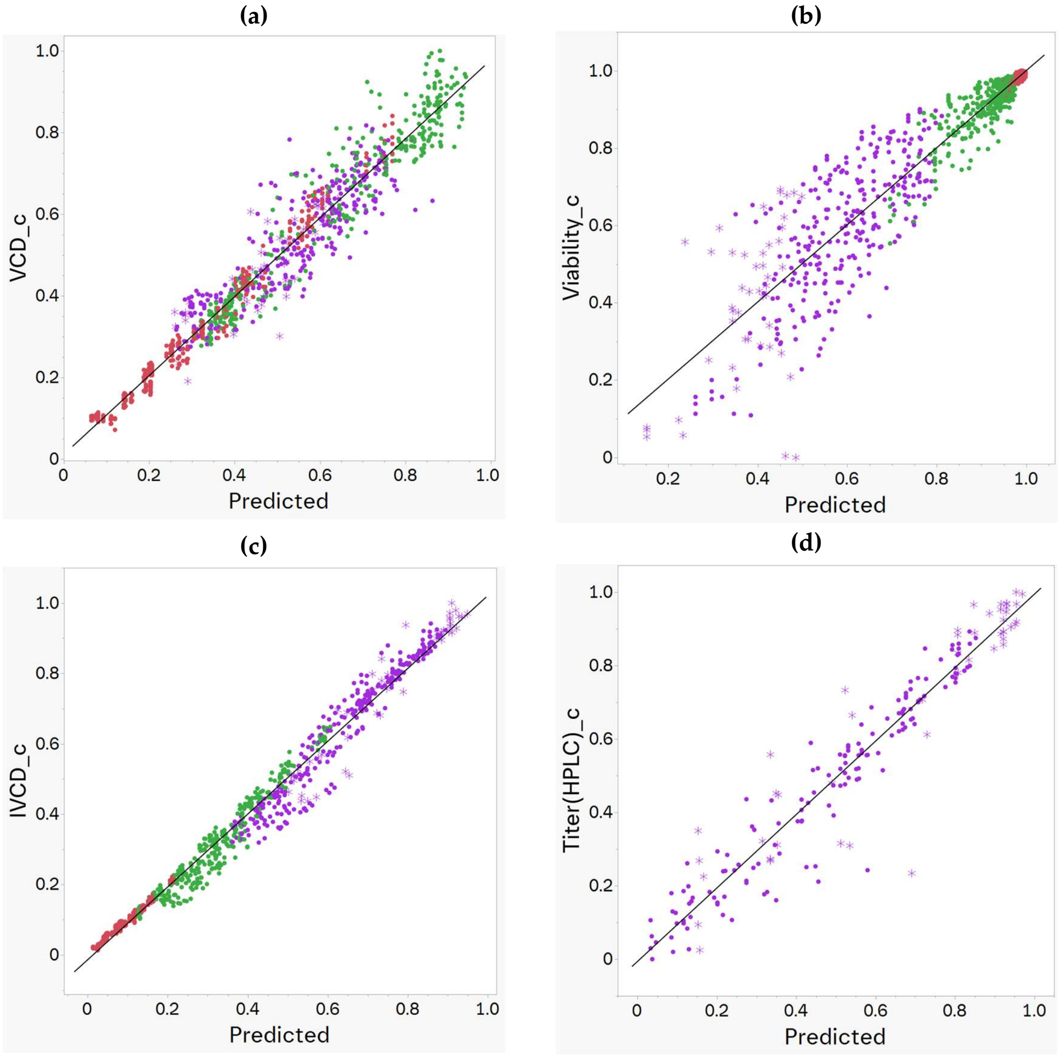

Figure S18. Actual vs predicted plots of the stage-wise models for the responses (**a**) viable cell density, (**b**) viability, (**c**) integral viable cell density, and (**d**) and titer RASE = Root average squared error, AAE = Average absolute error, stage 1 = red, stage 2 = green, stage 3 = purple, day 14 measurement = *

5.2. One-Factor-Multiple-Columns: Ordinary Least Squares Modeling

Table S4. Summary statistics for the one-factor-multiple-columns models for day 14 of the investigated responses

Response	Number of Parameters	R^2	R^2_{adj}	R^2_{pred}	RASE	RMSE	RMSE (PRESS)	Prob > F
VCD	9	0.859	0.834	0.802	0.044	0.049	0.053	<.0001
IVCD	16	0.987	0.981	0.974	0.022	0.027	0.031	<.0001
Viability	12	0.743	0.673	0.530	0.101	0.115	0.137	<.0001
Titer	14	0.967	0.955	0.932	0.054	0.063	0.077	<.0001

Table S5. Final one-factor-multiple-columns models. ASI = Across-stage-interaction 96

Response	Term(centered)	ASI	Estimate	Std Error	t Ratio	Prob>|t|	Lower 95%	Upper 95%
VCD	Intercept	non-ASI	0.468	0.007	64.925	0.000	0.453	0.482
VCD	T1	non-ASI	0.074	0.008	9.304	0.000	0.058	0.090
VCD	T2	non-ASI	0.042	0.008	5.195	0.000	0.026	0.059
VCD	T3	non-ASI	-0.048	0.008	-5.815	0.000	-0.064	-0.031
VCD	DO1	non-ASI	-0.036	0.009	-3.858	0.000	-0.054	-0.017
VCD	(T1-0.04167):(T2+0.16667)	ASI	-0.057	0.009	-6.367	0.000	-0.075	-0.039
VCD	(T1-0.04167):(T3+0.08333)	ASI	-0.029	0.009	-3.259	0.002	-0.047	-0.011
VCD	(T1-0.04167):DO1	non-ASI	0.051	0.010	4.942	0.000	0.030	0.073
IVCD	Intercept	non-ASI	0.798	0.010	81.295	0.000	0.778	0.818
IVCD	T1	non-ASI	0.198	0.004	44.734	0.000	0.189	0.207
IVCD	T2	non-ASI	0.034	0.005	7.570	0.000	0.025	0.043
IVCD	T3	non-ASI	-0.017	0.005	-3.735	0.001	-0.027	-0.008
IVCD	DO1	non-ASI	-0.035	0.005	-6.740	0.000	-0.046	-0.025
IVCD	DO2	non-ASI	-0.007	0.005	-1.385	0.175	-0.017	0.003
IVCD	DO3	non-ASI	-0.001	0.005	-0.102	0.919	-0.011	0.010
IVCD	(T1-0.04167):(T1-0.04167)	non-ASI	-0.051	0.010	-5.309	0.000	-0.070	-0.031
IVCD	(T1-0.04167):(T2+0.16667)	ASI	-0.036	0.005	-7.201	0.000	-0.046	-0.026
IVCD	(T1-0.04167):(T3+0.08333)	ASI	-0.010	0.005	-2.000	0.054	-0.020	0.000
IVCD	(T1-0.04167):DO1	non-ASI	0.018	0.006	3.096	0.004	0.006	0.031
IVCD	(T2+0.16667):DO1	ASI	-0.015	0.006	-2.565	0.015	-0.027	-0.003
IVCD	DO1:DO1	non-ASI	-0.020	0.008	-2.385	0.023	-0.037	-0.003
IVCD	(T1-0.04167):(DO3-0.02083)	ASI	0.015	0.005	2.652	0.012	0.003	0.026
IVCD	(DO2+0.02083):(DO3-0.02083)	ASI	-0.014	0.006	-2.418	0.021	-0.026	-0.002
Titer	Intercept	non-ASI	0.836	0.024	34.767	0.000	0.787	0.885
Titer	T1	non-ASI	0.276	0.011	26.116	0.000	0.254	0.297
Titer	T2	non-ASI	0.055	0.011	5.104	0.000	0.033	0.076

Titer	T3	non-ASI	0.019	0.011	1.765	0.086	-0.003	0.041
Titer	DO1	non-ASI	-0.099	0.012	-8.147	0.000	-0.124	-0.074
Titer	DO3	non-ASI	-0.014	0.012	-1.197	0.239	-0.039	0.010
Titer	(T1-0.04167):(T1-0.04167)	non-ASI	-0.126	0.023	-5.405	0.000	-0.173	-0.078
Titer	(T1-0.04167):(T2+0.16667)	ASI	-0.100	0.012	-8.260	0.000	-0.125	-0.075
Titer	(T2+0.16667):(T3+0.08333)	ASI	-0.030	0.013	-2.323	0.026	-0.056	-0.004
Titer	(T1-0.04167):DO1	non-ASI	0.072	0.014	5.238	0.000	0.044	0.100
Titer	DO1:DO1	non-ASI	-0.087	0.021	-4.207	0.000	-0.129	-0.045
Titer	(T3+0.08333):(DO3-0.02083)	non-ASI	0.031	0.014	2.296	0.028	0.004	0.059
Titer	DO1:(DO3-0.02083)	ASI	-0.030	0.014	-2.190	0.035	-0.058	-0.002
Viability	Intercept	non-ASI	0.308	0.037	8.331	0.000	0.233	0.383
Viability	T1	non-ASI	-0.103	0.019	-5.403	0.000	-0.141	-0.064
Viability	T2	non-ASI	-0.049	0.020	-2.489	0.017	-0.089	-0.009
Viability	T3	non-ASI	-0.086	0.020	-4.344	0.000	-0.126	-0.046
Viability	DO1	non-ASI	-0.046	0.022	-2.096	0.043	-0.091	-0.002
Viability	(T1-0.04167):(T1-0.04167)	non-ASI	0.116	0.041	2.798	0.008	0.032	0.200
Viability	(T1-0.04167):(T2+0.16667)	ASI	-0.045	0.021	-2.117	0.041	-0.088	-0.002
Viability	(T1-0.04167):(T3+0.08333)	ASI	-0.051	0.021	-2.413	0.021	-0.095	-0.008
Viability	(T1-0.04167):DO1	non-ASI	0.081	0.025	3.163	0.003	0.029	0.132
Viability	(T2+0.16667):DO1	ASI	0.080	0.025	3.196	0.003	0.029	0.130
Viability	(T3+0.08333):DO1	ASI	0.070	0.027	2.631	0.012	0.016	0.124

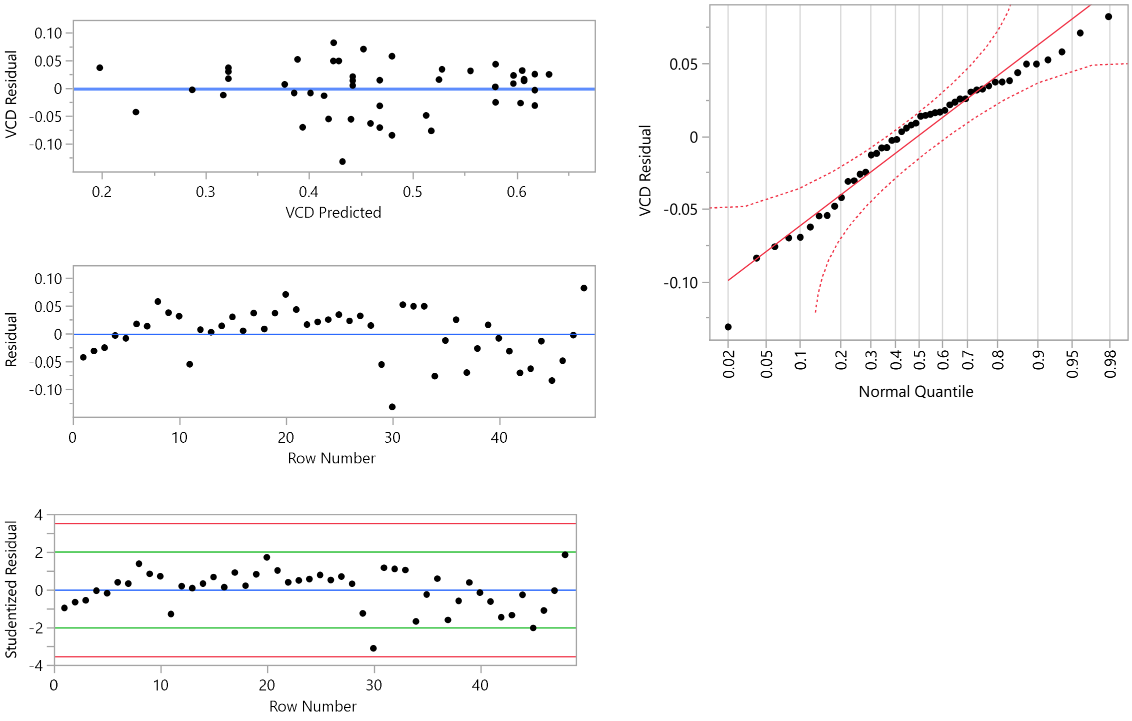

Figure S19. Residual plots for viable cell density (VCD) using one-factor-multiple-columns day 14 (OFMC-D14) modeling. Externally studentized residuals with 95% simultaneous limits (Bonferroni) in red, individual limits in green. Lilliefors confidence bounds in the normal quantile plot as dashed lines.

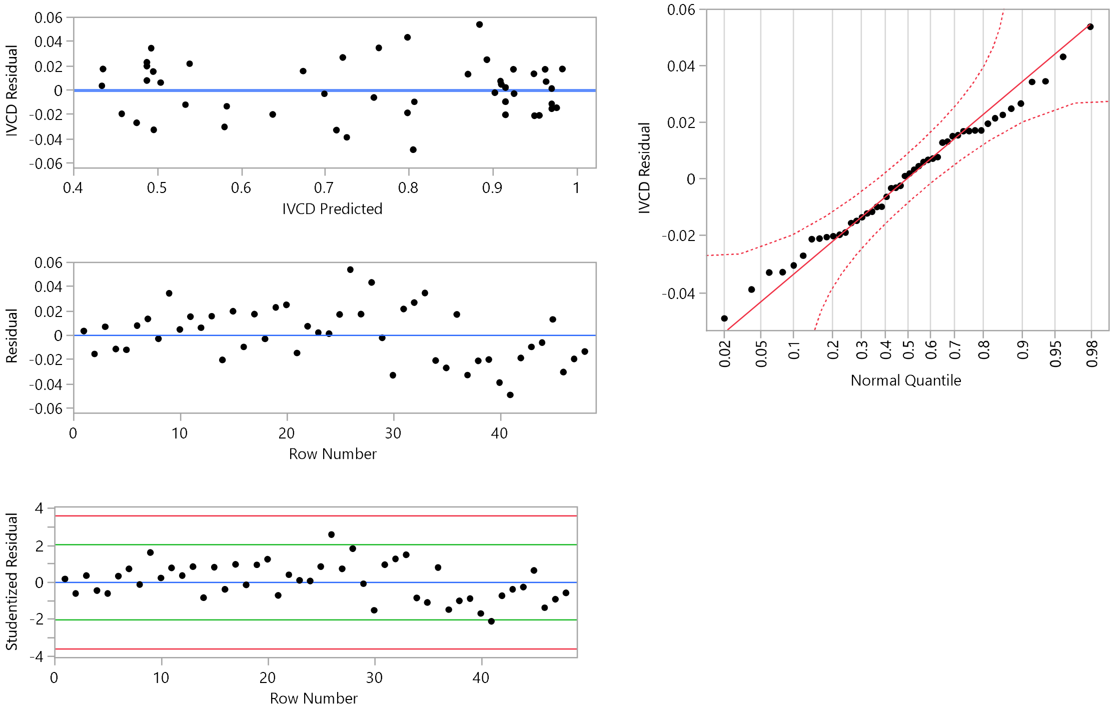

Figure S20. Residual plots for integral viable cell density (IVCD) using one-factor-multiple-columns day 14 (OFMC-D14) modeling. Externally studentized residuals with 95% simultaneous limits

(Bonferroni) in red, individual limits in green. Lilliefors confidence bounds in the normal quantile plot as dashed lines.

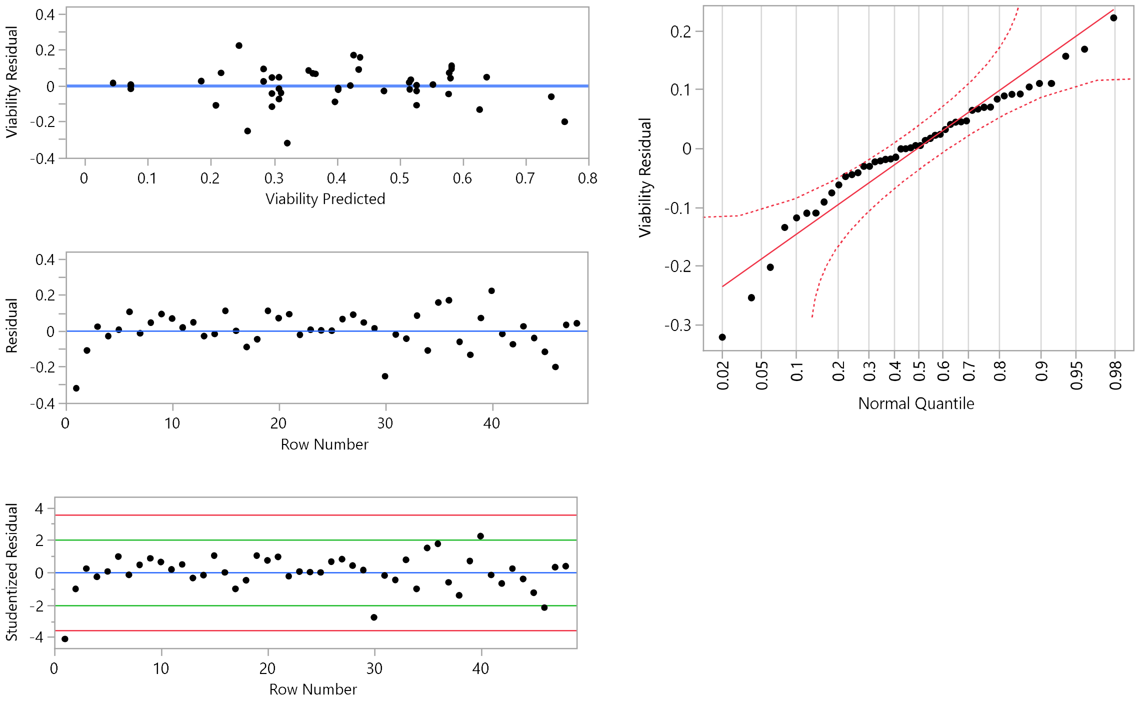

Figure S21. Residual plots for viability (Via) using one-factor-multiple-columns day 14 (OFMC-D14) modeling. Externally studentized residuals with 95% simultaneous limits (Bonferroni) in red, individual limits in green. Lilliefors confidence bounds in the normal quantile plot as dashed lines.

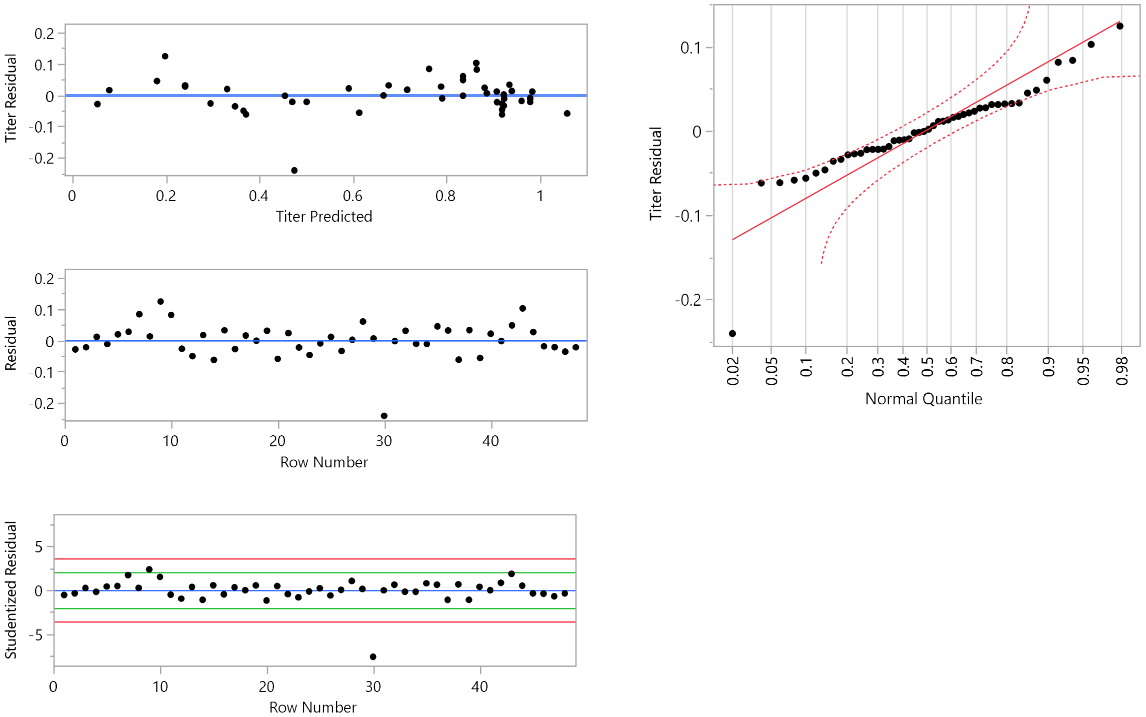

Figure S22. Residual plots for titer using one-factor-multiple-columns day 14 (OFMC-D14) modeling. Externally studentized residuals with 95% simultaneous limits (Bonferroni) in red, individual limits in green. Lilliefors confidence bounds in the normal quantile plot as dashed lines.

5.3. Visualization of Response Curves Colored based on iDoE Factors

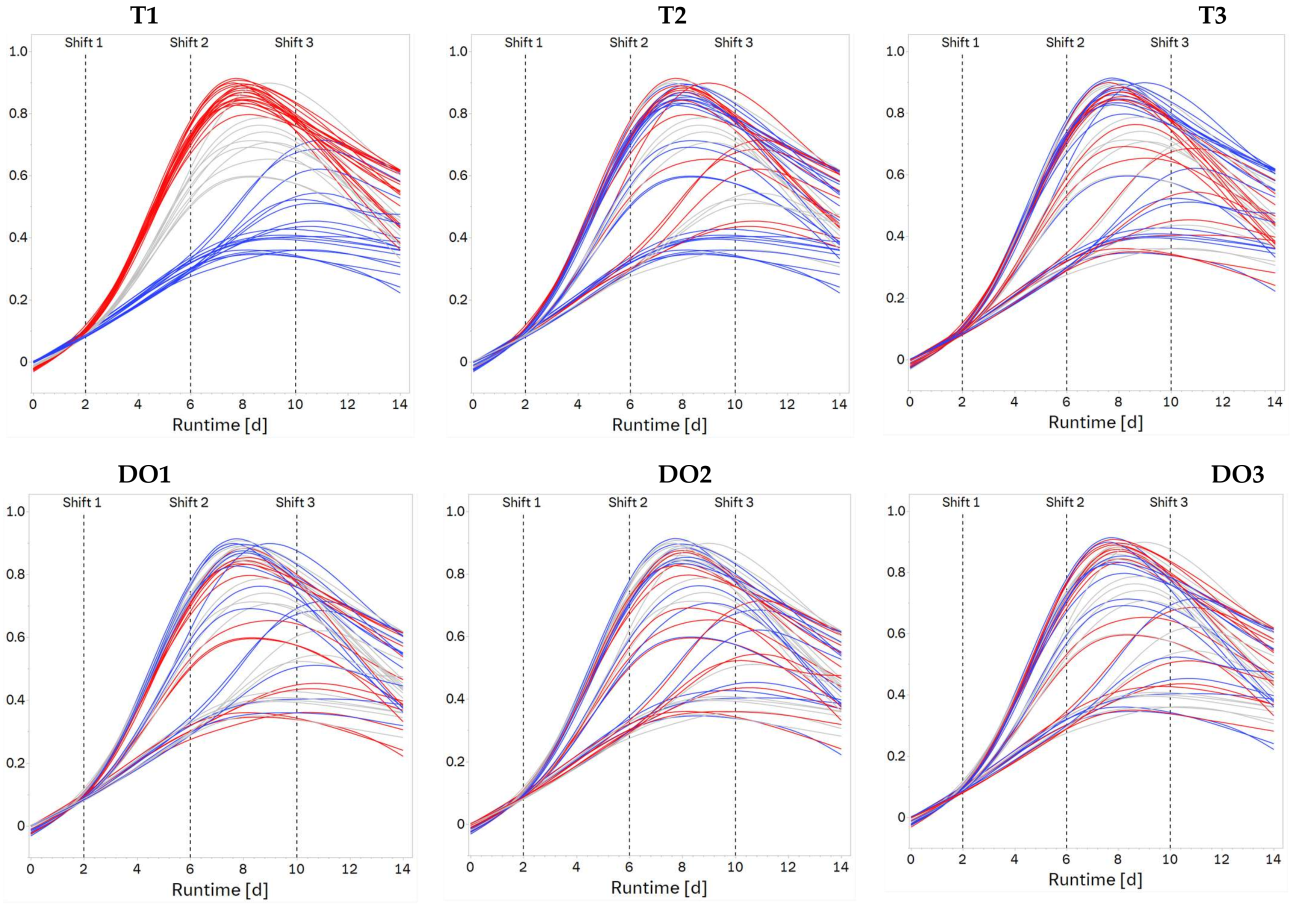

Figure S23. Viable cell density curves (smoothed) colorized by the iDoE factor settings of T1, T2, T3, DO1, DO2 and DO3. Red color indicates high level, grey indicates center level, blue indicates low level. Shift times of factor settings at day 2, 6 and 10 are indicated by dashed lines.

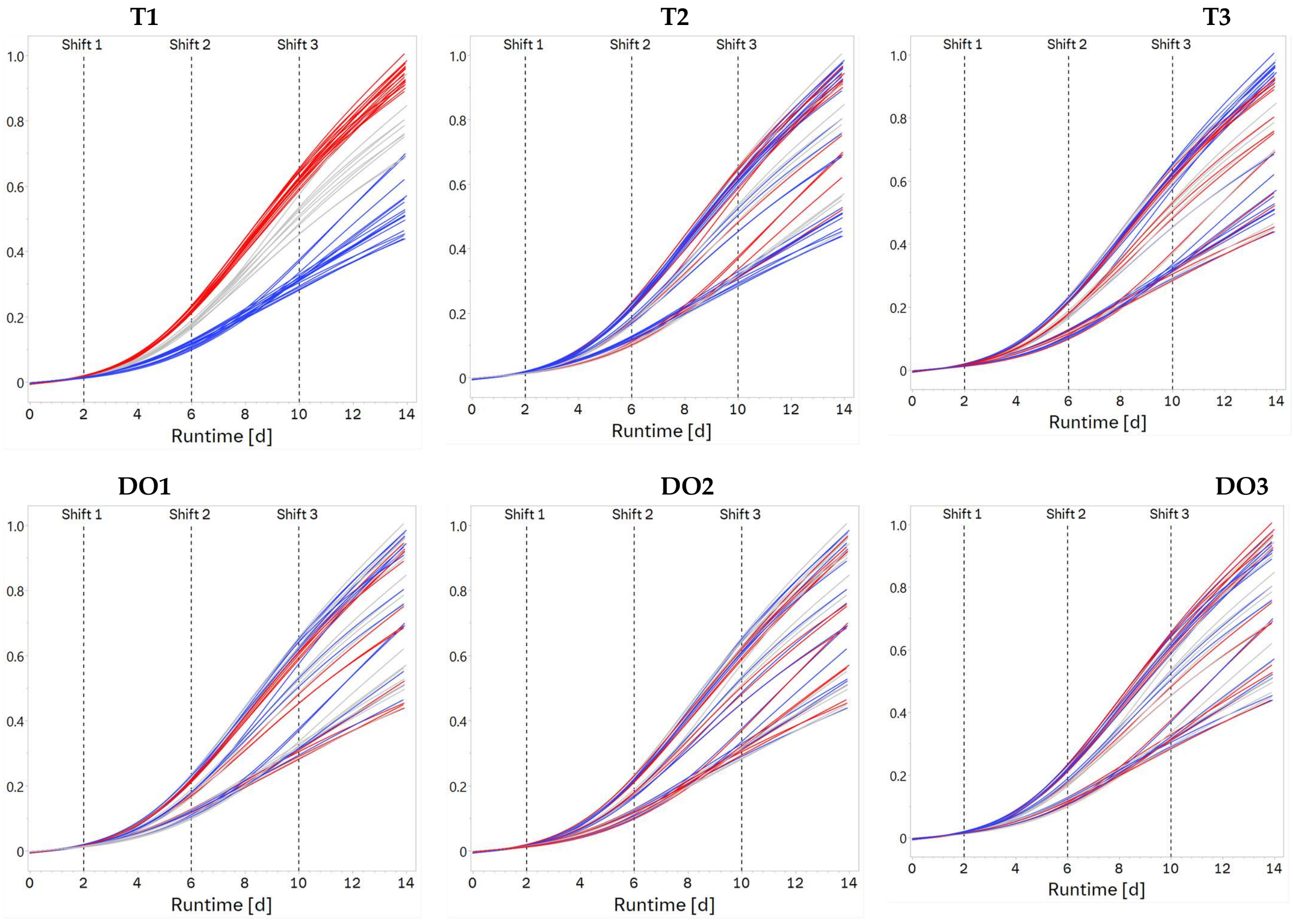

Figure S24. Integral Viable cell density curves (smoothed) colorized by the iDoE factor settings of T1, T2, T3, DO1, DO2 and DO3. Red color indicates high level, grey indicates center level, blue indicates low level. Shift times of factor settings at day 2, 6 and 10 are indicated by dashed lines.

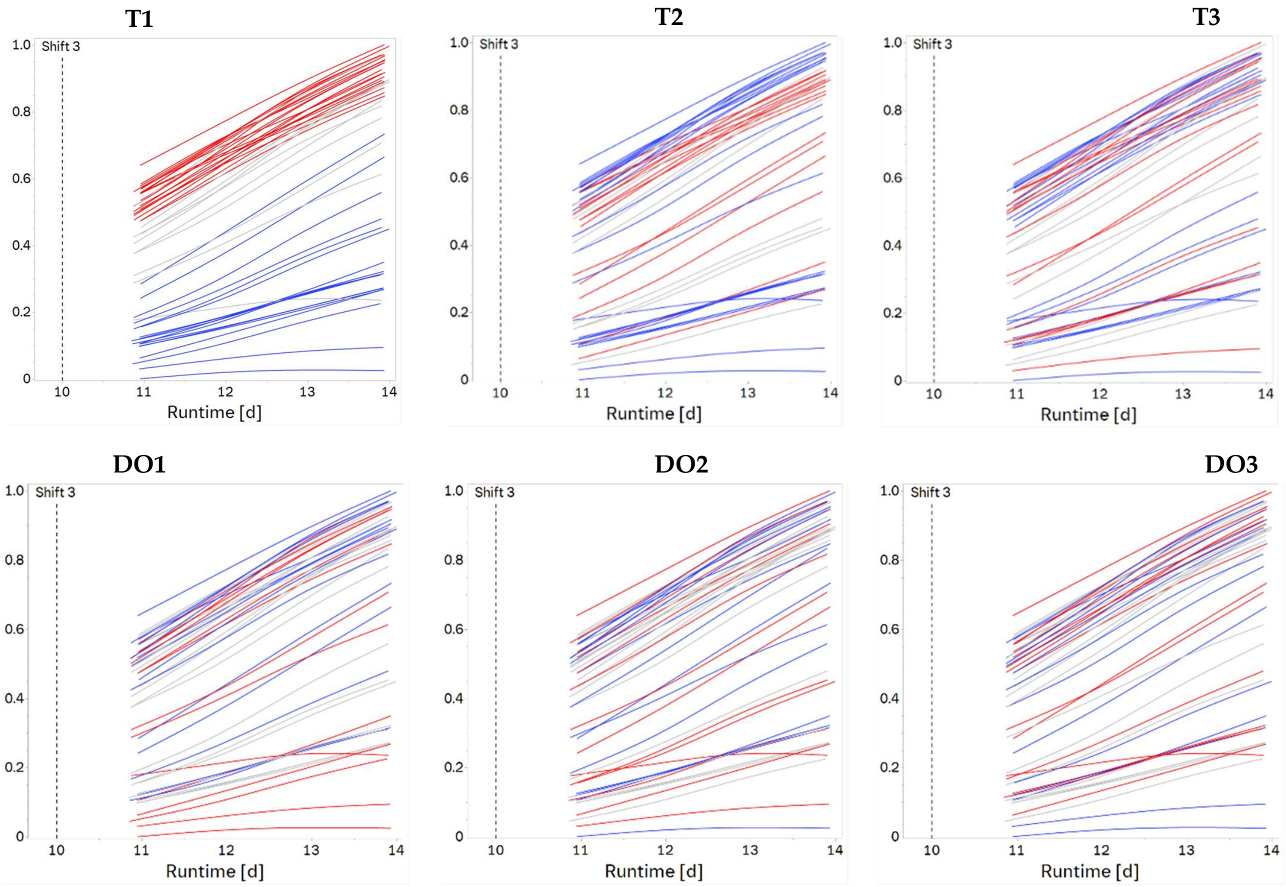

Figure S25. Titer curves (smoothed) colorized by the iDoE factor settings of T1, T2, T3, DO1, DO2 and DO3. Red color indicates high level, grey indicates center level, blue indicates low level. Shift times of factor settings at day 2, 6 and 10 are indicated by dashed lines.

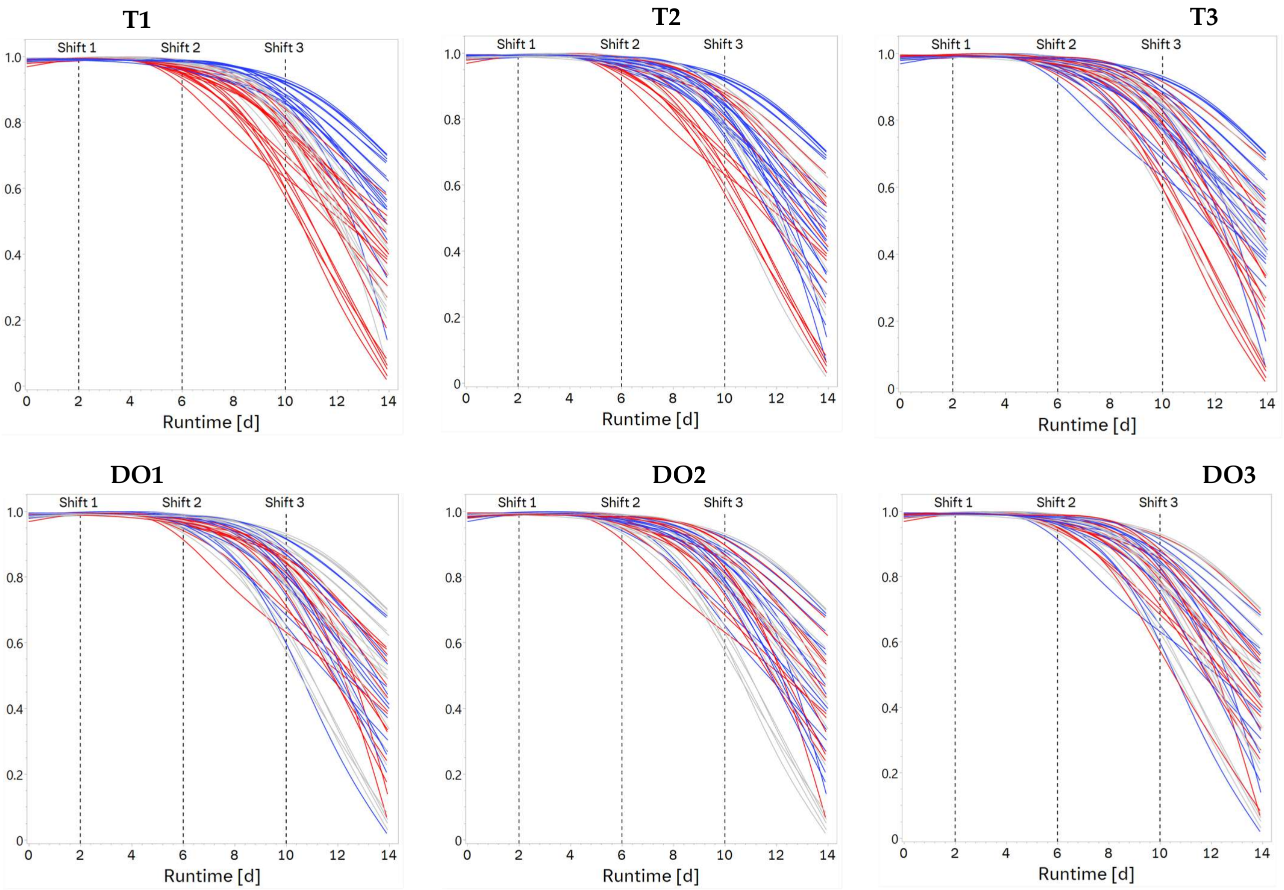

Figure S26. Viability curves (smoothed) colorized by the iDoE factor settings of T1, T2, T3, DO1, DO2 and DO3. Red color indicates high level, grey indicates center level, blue indicates low level. Shift times of factor settings at day 2, 6 and 10 are indicated by dashed lines.
